# Mutant Copper-Zinc Superoxide Dismutase (SOD1) Induces Protein Secretion Pathway Alterations and Exosome Release in Astrocytes: IMPLICATIONS FOR DISEASE SPREADING AND MOTOR NEURON PATHOLOGY IN AMYOTROPHIC LATERAL SCLEROSIS*[Fn FN2]

**DOI:** 10.1074/jbc.M112.425066

**Published:** 2013-04-16

**Authors:** Manuela Basso, Silvia Pozzi, Massimo Tortarolo, Fabio Fiordaliso, Cinzia Bisighini, Laura Pasetto, Gabriella Spaltro, Dario Lidonnici, Francesco Gensano, Elisa Battaglia, Caterina Bendotti, Valentina Bonetto

**Affiliations:** From the ‡ Dulbecco Telethon Institute,; the § Department of Neuroscience, and; the ¶ Bio-imaging Unit, Department of Cardiovascular Research, IRCCS-Istituto di Ricerche Farmacologiche Mario Negri, Via La Masa 19, 20156 Milano, Italy

**Keywords:** Amyotrophic Lateral Sclerosis (Lou Gehrig's Disease), Astrocytes, Exosomes, Proteomics, Superoxide Dismutase (SOD), Disease Spreading

## Abstract

Amyotrophic lateral sclerosis is the most common motor neuron disease and is still incurable. The mechanisms leading to the selective motor neuron vulnerability are still not known. The interplay between motor neurons and astrocytes is crucial in the outcome of the disease. We show that mutant copper-zinc superoxide dismutase (SOD1) overexpression in primary astrocyte cultures is associated with decreased levels of proteins involved in secretory pathways. This is linked to a general reduction of total secreted proteins, except for specific enrichment in a number of proteins in the media, such as mutant SOD1 and valosin-containing protein (VCP)/p97. Because there was also an increase in exosome release, we can deduce that astrocytes expressing mutant SOD1 activate unconventional secretory pathways, possibly as a protective mechanism. This may help limit the formation of intracellular aggregates and overcome mutant SOD1 toxicity. We also found that astrocyte-derived exosomes efficiently transfer mutant SOD1 to spinal neurons and induce selective motor neuron death. We conclude that the expression of mutant SOD1 has a substantial impact on astrocyte protein secretion pathways, contributing to motor neuron pathology and disease spread.

## Teaser

**Background:** The mechanism by which astrocytes contribute to disease progression in mutant SOD1 mouse models of ALS is not known.

**Results:** Mutant SOD1 astrocytes release mutant SOD1-containing exosomes that are toxic for motor neurons.

**Conclusion:** Astrocyte-derived exosomes may have a role in disease spreading and motor neuron pathology.

**Significance:** New therapeutic approaches should target exosomes to contain disease progression.

## Introduction

Amyotrophic lateral sclerosis (ALS)^5^ is a fatal, progressive neurological disease characterized by the specific degeneration of motor neurons in the cortex, brainstem, and spinal cord. The mechanisms involved in this selective degeneration are still not clear. In recent years, the interplay between motor neurons and glial cells, such as astrocytes, microglia, and oligodendrocytes, has been thoroughly investigated in mouse models of familial ALS-expressing forms of mutant copper-zinc superoxide dismutase (SOD1), as recently reviewed (1). These studies have clearly demonstrated that ALS is a non-cell-autonomous disease, where the interaction between motor neurons and other cell populations plays a role in motor neuronal degeneration and death.

Astrocytes, the most abundant glial cell type in the central nervous system, are responsible for major protective functions for motor neurons, such as releasing trophic factors and limiting motor neuron firing by clearing glutamate from the synaptic cleft. However, astrocytes can also adopt an activated state that is becoming increasingly appreciated as contributing to ALS. In patients and animal models of ALS, activated astrocytes display a decreased expression of the glutamate transporter that favors glutamate-induced excitotoxicity (2–4). Astrocyte cell cultures carrying mutant SOD1 release metabolites and/or proteins specifically toxic for motor neurons into the extracellular space (5–10). In addition, *in vivo* they appear to have an active role in pathology, and this has encouraged the use of these cells as a therapeutic target (11–14).

SOD1 is usually regarded as a cytosolic enzyme, but recently it has been reported extracellularly *in vitro*, under physiological and pathological conditions. A few studies have shown that SOD1 is secreted by different cells, including cultured astrocytes (15), motor neuron-like NSC-34 cells (16), and spinal cord cultures (17). More importantly, SOD1 has been detected in cerebrospinal fluid (CSF) of ALS patients with and without SOD1 mutations (18, 19). Turner *et al.* (16) considered the secretion of the mutant SOD1 from NSC-34 cells as beneficial, noting that the release of the mutant protein attenuates the formation of toxic intracellular inclusions and prolongs cell survival. However, Urushitani *et al.* (17) found that mutant SOD1, once secreted in association with chromogranins, is deleterious because it induces microgliosis and death of motor neurons in co-cultures. High-throughput technologies have started to identify the factors released by the astrocytes, aiming to uncover their various functions and the toxic molecules released (20–22). Numerous proteins have been listed, but the link with pathological events is still missing.

We therefore compared the proteome of the astrocytes from mice overexpressing mutant SOD1 (G93A SOD1), the best-characterized mouse model of familial ALS, with those from mice overexpressing human wild-type (WT) SOD1. The goal was to identify altered pathways induced by the expression of the mutant protein that may contribute to the disease. Interestingly, we observed alterations in the expression of proteins involved in the secretory pathways. We then proceeded to quantify the amount of proteins released by the two astrocyte populations and the content of the secretome (*i.e.* the profile of secreted proteins in the conditioned media). The amount of total secreted proteins by G93A SOD1 astrocytes was reduced, but levels of a selected number of proteins mostly known to be released by exosomes were increased (23).

Exosomes are vesicles generated by inward budding from the limiting membrane into the lumen of endosomes. In several hematopoietic and non-hematopoietic cells, multivesicular bodies fuse with the plasma membrane, and the internal vesicles are released into the extracellular space as exosomes (24). Exosomes are rich in proteins associated with the cytoskeleton, are linked to apoptosis, are involved in signal transduction, metabolism, and fusion of membranes, and seem also to contain mRNA and microRNA (25). Proposed functions of these vesicles include cell-cell signaling, removal of unwanted proteins, and transfer of pathogens between cells. It has been proposed that once released from the cell they might fuse with membranes of neighboring cells, transferring exosomal molecules from one cell to another. Some implications of these particles for neurodegenerative diseases are now emerging (26). We show for the first time that astrocytes overexpressing G93A SOD1 have increased exosome release, and astrocyte-derived exosomes readily transfer mutant SOD1 to spinal neurons and can induce selective motor neuron death.

## EXPERIMENTAL PROCEDURES

### 

#### 

##### Primary Astrocyte Cultures

Primary cultures were prepared from 15–16-day-old transgenic mouse embryos (Jackson Laboratories) expressing a high copy number of mutant (G93A) human SOD1 or human WT SOD1, as shown previously (27). Briefly, cortices were dissected and mechanically dissociated using a fire-polished glass Pasteur pipette in Hanks' balanced salt solution supplemented with glucose (33 mm). After centrifugation of the supernatant, the pellet was resuspended in astrocyte culture medium prepared with DMEM/F12 containing 2 mm l-glutamine, 33 mm glucose, 5 μg/ml gentamycin, 10% heat-inactivated horse serum, and seeded (500,000 cells/ml) onto 48- (only for co-culture preparation) or 6-well plates coated with 1.5 μg/ml poly-l-ornithine. Cells were grown at 37 °C in a humidified atmosphere with 5% CO_2_ and culture medium was first renewed after 6 days, then twice a week after washes in Hanks' balanced salt solution, 33 mm glucose to remove microglial cells, oligodendrocytes, and neurons. When the astrocytes reached confluence after 2–3 weeks in culture, proliferation was halted by treatment with 10 μm cytosine arabinoside for 3 days, and the cells were then ready for co-culture preparation. For all other experiments, astrocytes were washed six times with serum-free culture medium and incubated with phenol- and serum-free medium for 24 h at 37 °C and 5% CO_2_. Cells and media were then collected as described below. The absence of serum in the last passage before collection did not change cell viability (data not shown). Tissues expressing mutant human SOD1 were identified by PCR.

##### Protein Extraction from Astrocytes

For proteomic analysis, cells were resuspended in hot buffer (10 mm Tris-HCl, pH 7.5, 0.1% (v/v) Nonidet P-40, 0.1% (w/v) SDS). For immunoblotting analysis, cells were resuspended in hot buffer (1% (w/v) SDS), and DNA was fragmented using a syringe. Lysates were centrifuged at 10,000 × *g* for 15 min, and proteins were quantified with the BCA protein assay (Thermo Scientific).

##### Astrocyte-conditioned Media

Astrocyte-conditioned media for proteomic analysis were harvested and centrifuged at 200 × *g* (5 min), 1,000 × *g* (10 min) and 20,000 × *g* (25 min) to remove non-adherent cells and debris (preclearing). The media were dialyzed for 24 h in water with a 12 kDa cut-off membrane (SERVA), lyophilized, and stored at −20 °C. All steps up to lyophilization were carried out at 4 °C. For slot blot experiments, astrocyte-conditioned media were centrifuged at 12,000 × *g* for 5 min as preclearing, precipitated with four volumes of acetone for 2 h at 4 °C in agitation, and finally centrifuged at 15,000 × *g* for 10 min at 4 °C. Pellets were resuspended in hot lysis buffer (1% (w/v) SDS), and proteins were quantified by the BCA protein assay.

##### Isolation of Exosomes and Exosome-depleted Fractions

Precleared conditioned media, as described above, were ultracentrifuged at 100,000 × *g* for 1 h at 4 °C. The pellets (*i.e.* the exosomal fractions) were resuspended in hot lysis buffer (1% (w/v) SDS) for slot blot analysis or in the appropriate buffer for SDS-PAGE and two-dimensional gel electrophoresis, in phosphate-buffered saline (PBS) for electron microscopy or in Neurobasal medium for spinal neuron treatments (see below). Supernatants (*i.e.* the exosome-depleted fractions) were used for spinal neuron treatments or, after precipitation as described above, for biochemical analyses. The percentages of exosomal and non-exosomal proteins were calculated considering the total secreted proteins, measured by the BCA assay, as 100.

##### Two-dimensional Gel Electrophoresis

Protein samples were resuspended in 7 m urea, 2 m thiourea, 4% CHAPS and quantified by Bradford assay. Pools of 100 μg of astrocyte intracellular proteins or 30 μg of secreted proteins were used for two-dimensional gel electrophoresis analyses. At least six animals were used per pool. Three pools were analyzed on 7-cm IPG strips (GE Healthcare), pI 3–10, for astrocytes and pI 4–7 for conditioned media. For the astrocyte intracellular proteins, isoelectrofocusing was done according to the following schedule: 1 h at 100 V, 1 h and 30 min of a linear gradient to 500 V, 30 min of a linear gradient to 2,000 V and 30 min to 4,000 V, and 2 h at 8,000 V. A longer protocol was used for secreted proteins (50 V-h at 200 V, 1,375 V-h at 2,000 V, a linear gradient of 2,750 V-h to 3500 V, 7,000 V-h at 3,500 V, a linear gradient of 8,625 Vh to 8,000 V, and 32,000 V-h at 8000 V). Before SDS-PAGE, the IPG strips were equilibrated in 50 mm Tris-HCl, pH 8.8, 6 m urea, 30% glycerol, 2% SDS, and traces of bromphenol blue containing 0.1% (w/v) DTT for the first and 2.5% (w/v) iodoacetamide for the second equilibration step. SDS-PAGE was done using 4–12% NuPAGE gels (Invitrogen). After electrophoresis, gels were fixed in 50% methanol, 7% acetic acid for 30 min, stained with Sypro Ruby overnight, and washed with 10% methanol, 7% acetic acid for 10 min. Stained gels were scanned at 16-bit image (Molecular Imager FX, Bio-Rad) and the TIFF images generated were analyzed with Progenesis workstation software (PG240 v2006, Nonlinear Dynamics). Progenesis did spot detection, warping, and matching automatically. The results were carefully analyzed by the operator, and the protein spots significantly (*p* < 0.05, Student's *t* test) different were analyzed by mass spectrometry. We reported the ones with a -fold change of >1.5.

##### Protein Identification

Proteins were identified essentially as described previously (28). Briefly, protein spots were located and excised with an EXQuest^TM^ spot cutter (Bio-Rad). Spots were processed and gel-digested with trypsin, as described previously (29). Tryptic digests were concentrated and desalted using ZipTip pipette tips with C18 resin and a 0.2-ml bed volume (Millipore). Peptide mass fingerprinting was done on a ReflexIII^TM^ MALDI-TOF mass spectrometer (Bruker Daltonics) equipped with a SCOUT 384 multiprobe inlet and a 337-nm nitrogen laser using α-cyano-4-hydroxycinnamic acid as matrix, prepared as described previously (30). All mass spectra were obtained in positive reflector mode with a delayed extraction of 200 ns. The reflector voltage was 23 kV, and the detector voltage 1.7 kV. All of the other parameters were set for optimized mass resolution. To avoid detector saturation, low mass material (500 Da) was deflected. The mass spectra were internally calibrated with trypsin autolysis fragments. The mass spectra were obtained by averaging 150–350 individual laser shots and then automatically processed by the FlexAnalysis software, version 2.0, using the following parameters: the Savitzky Golay smoothing algorithm and the SNAP peak detection algorithm. Database searches were done using the Mascot software package available at the Matrix Science Web site, allowing up to one missed trypsin cleavage, carbamidomethylation of Cys and oxidation of Met as variable modifications, and a mass tolerance of 0.1 Da over all *Mus musculus* protein sequences deposited (SwissProt 2011_06, 529,056 sequences, 187,423,367 residues). A protein was considered identified if the following criteria were fulfilled: the probability-based MOWSE (31) score was above the 5% significance threshold for the database, and the spots excised from at least two different gels gave the same identification.

##### Western Blotting (WB)

Samples (15 μg) were separated in 12% SDS-polyacrylamide gels and transferred to polyvinylidene difluoride membranes (Millipore). Membranes were blocked with 3% (w/v) bovine serum albumin (BSA) (Sigma) and 0.1% (v/v) Tween 20 in Tris-buffered saline, pH 7.5, and incubated overnight at 4 °C with the following antibodies: rabbit polyclonal anti-human SOD1 (1:2,000; Millipore), anti-glial fibrillary acidic protein (GFAP) (1:5,000; Dako), anti-mitogen-activated protein kinase 1/2 (ERK1/2) (1:2,000; Cell Signaling), and anti-peptidyl-prolyl *cis-trans*-isomerase A (CypA) (1:2000, Millipore), mouse monoclonal anti-β actin (1:1,000; Santa Cruz Biotechnology, Inc., Santa Cruz, CA), and α-crystallin B chain (Cryab) (1:2,000; Stressgen). Densitometry was done with Progenesis PG240 v2006 software (Nonlinear Dynamics). The immunoreactivity of the different proteins was normalized to actin immunoreactivity or Ponceau Red staining (Fluka). Values were expressed as means ± S.E. Student's *t* test was used for statistical analysis.

##### Slot Blot

Proteins were directly loaded on preconditioned polyvinylidene difluoride membranes (Millipore) or nitrocellulose Trans-Blot transfer medium 0.2-μm (Bio-Rad) membranes. Each sample was deposited on the membrane by vacuum filtration. Aliquots (2 μg) of samples from G93A SOD1 or WT SOD1 astrocytes were loaded on the membrane, which was probed with the mouse monoclonal anti-nitrotyrosine antibody (1:1,000; HyCult Biotechnology). One-tenth or one-twentieth of the total volume of secreted, exosomal-enriched or non-exosomal protein fractions was used to detect specific protein levels. Media were collected from equally plated astrocytes, as measured by total protein content. The antibodies anti-human SOD1 (1:500; Millipore), anti-CypA (1:1,000; Millipore), and rabbit monoclonal anti-valosin-containing protein (VCP)/p97 (1:50,000; Epitomics) were incubated overnight at 4 °C to reveal the immunoreactivity in secreted proteins and exosome-enriched fractions. The mouse monoclonal anti-flotillin-1 (clone 18, BD Transduction Laboratories) was used diluted 1:500 to detect the specificity and amount of exosomal fraction preparation. Blots were probed with anti-mouse or anti-rabbit HRP-conjugated antibody (1:5,000; Santa Cruz Biotechnology, Inc.) and visualized by Immobilon Western Chemiluminescent HRP substrate (Millipore) on a ChemiDoc XRS system (Bio-Rad). Densitometry was done with Progenesis PG240 v2006 software (Nonlinear Dynamics). The immunoreactivity was normalized to the actual amount of proteins loaded on each slot on the membrane, as detected after Coomassie or Sypro Ruby blot (Bio-Rad) staining. Values were expressed as means ± S.E. Student's *t* test was used for statistical analysis.

##### Primary Spinal Neuron Cultures and Treatments

Non-transgenic spinal neurons were prepared from spinal cords of day 14 embryos, as described (32). Briefly, the whole spinal cord was dissected away, and tissues were mechanically dissociated in Hanks' balanced salt solution, 33 mm glucose, and the cell suspension was centrifuged onto a 4% BSA cushion. The pellet was resuspended in Neurobasal medium (Invitrogen) supplemented with 2 mm l-glutamine, 33 mm glucose, 5 μg/ml gentamycin, 1 ng/ml brain-derived neurotrophic factor, and hormonal mix (25 μg/ml insulin, 10 μg/ml putrescine, 30 nm sodium selenite, 2 μm progesterone, 100 μg/ml apo-transferrin). Cells were plated on glass coverslips, coated with 15 μg/ml poly-l-ornithine and 2 μg/ml natural mouse laminin, and exposed overnight to 0.5 ml of (i) Neurobasal medium resuspension of exosomes obtained from 2.5 ml of conditioned medium from astrocytes, (ii) exosome-depleted supernatants, or (iii) non-processed conditioned medium.

##### Immunoelectron Microscopy on Isolated Exosomes and Spinal Neuron Cultures

A drop of 5 μl of purified exosomes in PBS was placed to dry at room temperature on a 100-mesh Formvar/carbon-coated copper grid (EMS, Hatfield, PA) and fixed with 4% paraformaldehyde and 2% glutaraldehyde in 0.15 m HEPES buffer (pH 7.4) for 30 min. Exosomes were then incubated with rabbit polyclonal anti-human SOD1 (1:100; Millipore) overnight at 4 °C, followed by 10-nm colloidal gold-conjugated-protein A incubation (Cell Microscopy Center, Utrecht, The Netherlands) for 30 min. Grids were finally counterstained and embedded in a mixture of methylcellulose and uranyl acetate and observed with an energy filter transmission electron microscope (LIBRA® 120, Zeiss) equipped with a YAG scintillator slow scan CCD camera (Zeiss). For spinal neuron analysis, cells were cultured on glass coverslips of 24-well multiwell plates for 5 days and exposed to exosomes or exosome-depleted fractions for 24 h. After incubation, medium was discarded, and cells were prefixed for 30 min at room temperature with 4% paraformaldehyde and 0.1% glutaraldehyde in 0.2 m Hepes (pH 7.4) and fixed at room temperature in 4% paraformaldehyde in 0.15 m Hepes, pH 7.4, for 30 min. Neurons were incubated for 30 min in blocking solution (1% BSA in PBS) and then overnight at 4 °C with rabbit polyclonal anti-human SOD1 (Millipore) diluted 1:100 in blocking solution, followed by 10-nm colloidal gold-conjugated-protein A for 30 min at room temperature. After postfixation with 1% glutaraldehyde in 0.15 m Hepes and incubation with 1% osmium in 0.1 m phosphate buffer, pH 6.8–7.4, on ice, cells were incubated 5 min at room temperature with a saturated solution of thiocarbohydrazide followed by 1.5% ferrocyanide and 1% osmium in 0.1 m phosphate buffer for 30 min. Neurons, still attached on the coverslip, were then counterstained with 0.5% uranyl acetate overnight at 4 °C, dehydrated in graded series of ethanol, and embedded in Epoxy medium (Epon 812 Fluka, Sigma-Aldrich). Thereafter, the coverslip was removed from the well of multiwell plate and, to allow the transfer of cells from coverslip to resin block, was placed bottom-up on a disposable flat embedding mold (catalog no. 70906-10, Electron Microscopy Sciences) prefilled with epoxy resin and polymerized at 60 °C for 72 h. After removing the glass coverslip using 40% hydrofluoric acid, the resin block with neurons on the top was trimmed to obtain a small pyramid suitable for ultrathin (55–60 nm thick) sectioning with a Leica EM UC6 ultramicrotome. Sections were then collected on 100-mesh Formvar/carbon-coated grids and examined with an energy filter transmission electron microscope equipped with a YAG scintillator slow scan CCD camera (Zeiss).

##### Spinal Neuron-Astrocyte Co-cultures and Treatments

Spinal neurons obtained as reported above were co-cultured in a 48-well Nunc multiwell plate with a pre-established cortical astrocyte confluent layer, prepared as described above, using neuron culture medium enriched with 10% heat-inactivated horse serum containing 10 μm cytosine arabinoside to avoid spinal glia proliferation. Non-transgenic spinal neurons (250,000 cells/well, as counted in a Burker's chamber) were plated on non-transgenic astrocytes using 200 μl of (i) Neurobasal medium (untreated) or Neurobasal medium resuspension of (ii) exosomes obtained from 200 μl of conditioned medium (exosomes 1×), (iii) exosomes obtained from 1 ml of conditioned medium (exosomes 5×), or (iv) exosomes obtained from 2 ml of conditioned medium (exosomes 10×) from astrocytes for each well. Co-cultures of non-transgenic spinal neurons plated on transgenic astrocytes were used as control. After spinal neuron plating, co-cultures were maintained for 6 days in culture, fixed for 30 min with 4% paraformaldehyde, rinsed with 0.01 m PBS, pH 7.4, and stored at 4 °C for subsequent analyses.

##### Motor Neuron Viability Assay

Motor neuron survival in astrocyte-spinal neuron co-cultures was determined by double immunocytochemistry for SMI32 (motor neuron marker) and NeuN (nuclear neuron marker), as described (32). Motor neuron survival was expressed as the ratio of the number of motor neurons (SMI32-positive cells) to the total neurons in the well (NeuN-positive cells). Wells were analyzed by an Olympus camera on a motorized microscope (Olympus, Tokyo, Japan). A reproducible grid of 9 × 9 frames (10× enlargement) was created, and 20 frames were acquired at 488 nm for NeuN and 648 nm for SMI32. NeuN-positive cells were automatically counted by ImageJ software (National Institutes of Health), and SMI32-positive cells were counted manually with Cell[caret]P software (Olympus) that identifies motor neurons as cells with extensive dendritic arborization and cell bodies of ≥20 μm.

## RESULTS

### 

#### 

##### Proteomic Analysis of Primary Astrocyte Cultures Expressing Human G93A SOD1 Reveals Down-regulation of Proteins Involved in Secretory Pathways

**FIGURE 1.**
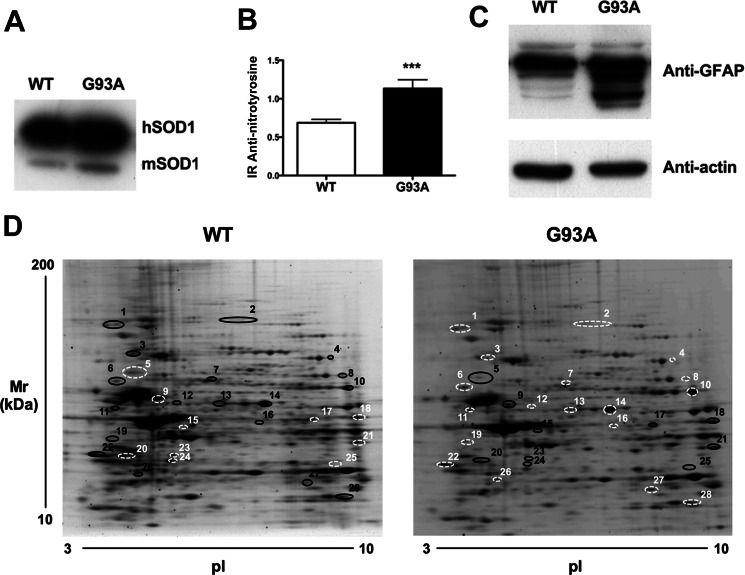
**Characterization of primary astrocyte cultures derived from G93A and WT SOD1 mice and protein differential expression.** *A*, cultures derived from G93A and WT SOD1 mice express similar amounts of human SOD1 (*hSOD1*) protein. A representative WB of human SOD1 levels and murine SOD1 (*mSOD1*) is also shown. *B*, slot blot for nitrotyrosine residues. Mutant SOD1 expression increases the levels of nitrotyrosine. Nitrotyrosine immunoreactivity (*IR*) was normalized to the total protein loaded, as measured after Coomassie staining. Values are the means ± S.E. (*error bars*) (*n* = 9). *Asterisks* indicate a G93A sample mean significantly higher (*p* < 0.001) than the WT sample mean, Student's *t* test. *C*, WB for GFAP in WT and G93A SOD1 astrocyte primary cultures. G93A SOD1 astrocytes present an increased number of GFAP fragments. *D*, 100 μg of proteins were loaded onto IPG strips (pI 3–10). Two-dimensional gel electrophoresis maps were stained with Sypro Ruby and analyzed with Progenesis software. The comparison revealed that the expression of mutant SOD1 is sufficient to alter the proteomic profile of the astrocytes (see Table 1). *Black circles*, up-regulated proteins; *white circles*, down-regulated proteins; *spot numbers*, proteins listed in Table 1.

**FIGURE 2.**
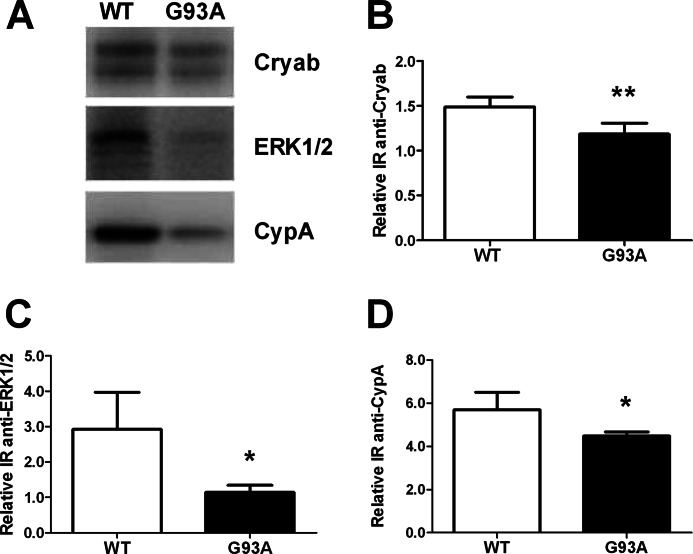
**Western blots of three differentially expressed proteins as validation of the proteomic analysis.** *A–D*, representative WB for Cryab, ERK1/2, and CypA and quantification. Samples were 3-well pools of 6-well dishes from four separate primary astrocyte culture preparations. Ponceau Red staining was used as loading control. Values are relative immunoreactivities (*IR*) and are means ± S.E. (*error bars*) (*n* = 8). All of the proteins were significantly down-regulated (*, *p* < 0.05; **, *p* < 0.01) in astrocytes expressing mutant SOD1 in comparison with WT SOD1 controls (Student's *t* test).

To investigate the role of the glia in ALS, we compared the proteome of primary astrocyte cultures from mice overexpressing similar amounts of human WT SOD1 and mutant G93A SOD1 (Fig. 1*A*). First we measured the levels of nitrotyrosine and GFAP as molecular markers of cellular alterations (10, 33). We detected increased levels of nitrotyrosine (Fig. 1*B*) and GFAP fragments (Fig. 1*C*) in G93A SOD1-expressing cells. GFAP fragments may be due to calpain activation, as reported previously in human ALS spinal cord (34). This confirms that the expression of mutant SOD1 was sufficient on its own to induce differences in astrocytes. We then did a two-dimensional gel electrophoresis-based proteomic analysis (Fig. 1*D*) where three different pools of astrocytes were run for each genotype. Image analysis by the Progenesis software detected 332 matched spots in WT and G93A SOD1 astrocyte maps; 30 spots were more present in the G93A SOD1 samples, 39 spots in the WT SOD1 samples. We then identified by MALDI-TOF mass spectrometry the proteins differentially expressed in the various samples and reported the ones with significant -fold change >1.5 (Table 1 and supplemental Table S1). Finally, we validated the analysis by WB of three representative proteins, Cryab, ERK1/2, and CypA (Fig. 2). Surprisingly, the majority were down-regulated in G93A SOD1 samples (Table 1), particularly proteins that are important in secretory pathways, such as those of the endoplasmic reticulum (ER) (HSP90B1 (heat shock protein 90-kDa β member 1), GRP78 (78-kDa glucose-regulated protein), and protein-disulfide isomerase) and those involved in vesicle trafficking (RabGDI (Rab GDP dissociation inhibitor β) and RhoGDI (Rho GDP-dissociation inhibitor 1)) (35, 36). In addition, alteration of a number of cytoskeleton-associated proteins, such as actin, vimentin, and GFAP, may reduce exocytotic vesicle mobility (37). Considering that one of the main properties of astrocytes is to release neuroactive substances extracellularly, we then investigated the conditioned media of mutant SOD1 astrocytes to see whether the alterations to proteins in secretory pathways involved deficient and/or altered protein secretion.

**TABLE 1:** **Differentially expressed proteins in primary astrocytes derived from G93A and WT SOD1 mice** Proteins were identified by MALDI-TOF mass spectrometry as reported under “Experimental Procedures” and in Table S1.

Spots*^a^*	Uniprot*^b^*	Protein name	Over*^c^*	Under*^d^*
			*-fold*	*-fold*
**Cytoskeleton-associated**				
2	Q8BTM8	Filamin-A		2.3*^e^*
5	P20152	Vimentin	1.5	
9	P03995 + P20152	GFAP + vimentin	4.0	
11	P20152	Vimentin		3.7*^e^*
15	P60710	Actin	1.6	
19	P58771	Tropomyosin-1 α chain		1.8*^e^*
20	P68372	Tubulin β-2C chain	2.1	
23	P60710	Actin	1.7	

**Energy metabolism**				
4	P40142	Transketolase		1.7*^e^*
8	P52480	Pyruvate kinase isozyme M2		2.5*^e^*
10	Q03265	ATP synthase subunit α		3.1*^e^*
12	Q9CZ13	Ubiquinol-cytochrome *c* reductase complex core protein 1		3.1*^e^*
14	P17182	α-Enolase		1.6*^e^*
17	P05063	Fructose-bisphosphate aldolase C	2.1	
18	P05064	Fructose-bisphosphate aldolase A	1.7	
25	Q8BH95	Enoyl-CoA hydratase	1.8	

**Endoplasmic reticulum**				
1	P08113	Heat shock protein 90 kDa β member1 (HSP90B1)		1.6*^e^*
3	P20029	78-kDa glucose-regulated protein (GRP78)		1.5*^e^*
6	P09103	Protein-disulfide isomerase (PDI)		1.5*^e^*

**Vesicle trafficking**				
13	Q61598	Rab GDP dissociation inhibitor β (RabGDI)		1.5*^e^*
26	Q99PT1	Rho GDP-dissociation inhibitor 1 (RhoGDI)		1.6*^e^*

**Signaling**				
16	P63085	Mitogen-activated protein kinase1 (ERK2)		2.2*^e^*
22	P62259	14-3-3 protein ϵ		1.5*^e^*
7	O08553	Dihydropyrimidinase-related protein2 (DRP-2)		2.3*^e^*

**Chaperones**				
27	P23927	α-Crystallin B chain (Cryab)		1.5*^e^*
28	P17742	Peptidyl-prolyl *cis-trans*-isomerase A (CypA)		1.6*^e^*

**Others**				
21	Q60932	Voltage-dependent anion-selective channel protein 1	1.9	
24	P67778	Prohibitin	1.6	

*^a^* Spot numbers (see Fig. 1*D*).

*^b^* Entry from the Uniprot Knowledgebase database.

*^c^* Overexpressed proteins in G93A SOD1 astrocytes; values are volume -fold changes (G93A/WT).

*^d^* Underexpressed proteins in G93A SOD1 astrocytes; values are volume -fold changes (WT/G93A). *Proteins identified in exosomes (ExoCarta database) (23).

##### Mutant SOD1 Astrocytes Globally Release a Lower Amount of Proteins but a Larger Proportion of Proteins through Exosomes

**FIGURE 3.**
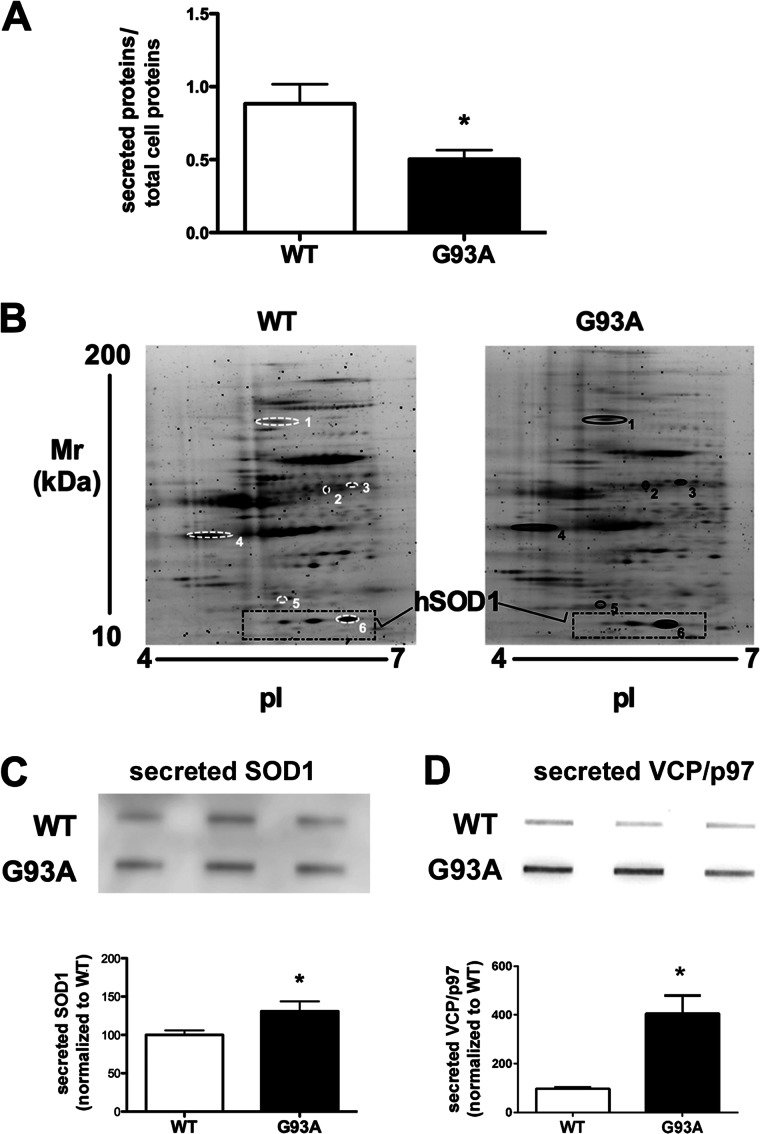
**G93A SOD1 astrocytes released a lower amount of proteins in the media.** *A*, media were collected from similar numbers of cells expressing WT or G93A SOD1, and secreted proteins were prepared as described for slot blot experiments and quantified by BCA protein assay. Protein quantification was performed in at least seven samples per group. Each sample was a 3-well pool. Values are the amounts of secreted proteins normalized to total cell proteins (secreted proteins/total cell proteins) and are means ± S.E. (*error bars*). An *asterisk* indicates statistical significance (*p* < 0.05), Student's *t* test. *B*, for two-dimensional gel electrophoresis maps (*n* = 3 for each genotype), the same amount of secreted proteins (30 μg) were loaded on IPG strips (pI 4–7). The gel maps were stained with Sypro Ruby and compared by computerized image analysis. Six proteins were differentially secreted by astrocytes expressing mutant SOD1 and controls. *Spot numbers* refer to proteins listed in Table 2. *C* and *D*, slot blot and relative quantification of SOD1 (*C*) and VCP/p97 (*D*) released by the astrocytes confirmed the proteomic analysis and showed that the total level of the extracellular proteins were higher than in control conditions. The astrocytes were plated in 6-well dishes, and equal volumes of conditioned media from 3-well pools were used for the slot blot analysis. Values are immunoreactivities and are means ± S.E. (*n* = 3) normalized to WT, set as 100. An *asterisk* indicates statistical significance (*p* < 0.05), Student's *t* test.

**FIGURE 4.**
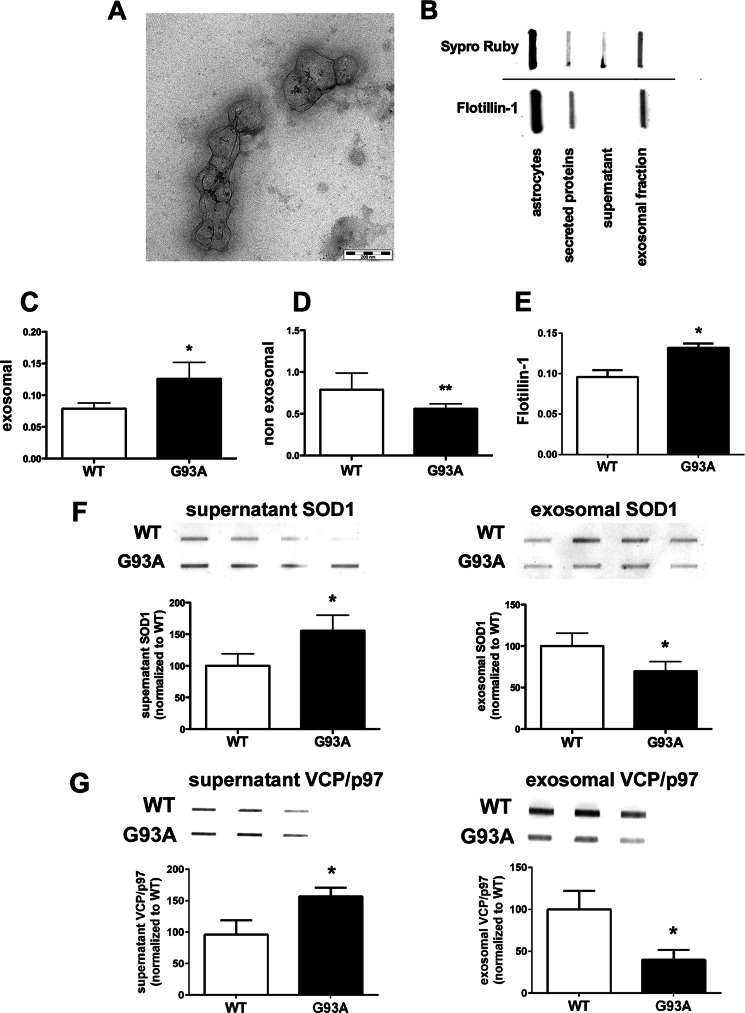
**WT and G93A SOD1 are released by the astrocytes, and different amounts are present in the exosomes.** *A*, electron microscopy analysis of purified membrane vesicles with typical exosomal shape and dimension (diameter ranging from 80 to 140 nm; *scale bar*, 200 nm). *B*, slot blot of astrocyte protein lysate, secreted proteins, and ultracentrifuged proteins (supernatant and exosomal fractions). An exosomal marker, flotillin-1, is present in whole lysate, in unpurified secreted proteins, and in the exosomal fraction but not in the supernatant. *C* and *E*, G93A SOD1 expression increases exosomal proteins. Protein quantification of the supernatant and exosomal fractions was done by BCA protein assay in at least seven samples per genotype. Each sample was a 3-well pool. Values are the amounts of non-exosomal or exosomal proteins normalized to total astrocytic proteins (non-exosomal or exosomal proteins/total astrocytic proteins) and are means ± S.E. (*error bars*) *Asterisks* indicate statistical significance (*, *p* < 0.05; **, *p* < 0.01), Student's *t* test. *D*, flotillin-1 was measured by slot blot in WT and G93A SOD1 astrocyte-conditioned media. Anti-flotillin-1 immunoreactivity was normalized to the total protein loaded, as measured after Sypro Ruby blot staining. Values are means ± S.E. (*n* = 3). The *asterisk* indicates a G93A sample mean significantly higher (*p* < 0.05) than the WT sample mean, Student's *t* test. *F* and *G*, slot blot for SOD1 and VCP/p97 in the exosomal and non-exosomal (supernatant) fractions. Equal volumes of exosomal or non-exosomal fractions were used to measure protein levels in the G93A SOD1 and WT SOD1 conditions. Each sample was taken from a 3-well pool. Values are immunoreactivities and are means ± S.E. (*n* = 4) normalized to WT, set as 100. An *asterisk* indicates statistical significance (*p* < 0.05), Student's *t* test.

In the conditioned media from WT and mutant SOD1 astrocytes, G93A SOD1 astrocytes globally secreted 43% fewer proteins than WT SOD1 astrocytes (Fig. 3*A*), confirming an impairment of protein secretion. In the subsequent differential analysis of the secretome of G93A SOD1 and WT SOD1 astrocytes by two-dimensional gel electrophoresis-based proteomics, surprisingly, the protein patterns of the two conditions were very similar except for six spots (Fig. 3*B*). All were more present in the G93A SOD1 media, and these included human SOD1, with one of the highest -fold changes, and VCP/p97 (Table 2 and supplemental Table S1). The two-dimensional poststained proteomic approach for secreted proteins has high inherent variability because of difficulties in quantification of highly glycosylated proteins (38). However, the increasing total levels of mutant SOD1 and VCP/p97 in astrocyte-conditioned media were confirmed by slot blot (Fig. 3, *C* and *D*). Interestingly, only two of the differentially present proteins, ERp57 (protein-disulfide isomerase A3) and SPARC (secreted protein acidic and rich in cysteine), are classically secreted proteins and have a predicted signal peptide (39). Four of the six (VCP/p97, DRP-2 (dihydropyrimidinase-related protein 2), ERp57, and SOD1) have been identified in exosomes of various cell types (see the database at the ExoCarta Web site) (23, 40). We then hypothesized that their secretion by astrocytes may also occur through exosomes and wondered whether mutant SOD1 was inducing exosome secretion in astrocytes. We isolated and characterized exosomes from mutant and WT SOD1 astrocyte-conditioned media (Fig. 4, *A* and *B*). Electron microscopy showed that exosomal fractions contained vesicular elements of the expected size (80–140 nm) and characteristic morphology (41) (Fig. 4*A*). Accordingly, only the exosome fractions were specifically enriched in exosome marker flotillin-1 (42) (Fig. 4*B*). When we quantified the total amount of proteins associated with this fraction compared with controls (Fig. 4*C*), we found a significantly greater level (37%) of proteins in G93A SOD1-derived exosome fractions than in WT fractions. We noted a similar increase (38%) in flotillin-1 exosome marker in the conditioned media of G93A SOD1 astrocytes (Fig. 4*D*). Because flotillin-1 is a scaffolding protein of lipid raft microdomains that forms the exosomal membrane, we hypothesized an up-regulation in vesicle release rather than enrichment of proteins per single exosome. In agreement with the results of the unprocessed conditioned media (Fig. 3*A*), the decrease in the amount of proteins in the corresponding G93A SOD1-derived exosome-depleted fractions was maintained (Fig. 4*E*). This was expected, because the majority of the secreted proteins (91% in WT SOD1 and 82% in G93A SOD1 cells) are recovered in exosome-depleted fractions. Finally, we analyzed the amount of SOD1 in the two fractions. In WT SOD1 samples, 66% of total secreted SOD1 was associated with the exosome-depleted fraction, and 34% was recovered in the exosomal fraction. In G93A SOD1 samples, the mutant protein in the exosomal fraction was 37% lower than in the WT (Fig. 4*F*). The pattern was overall similar for VCP/p97 (Fig. 4*G*). In conclusion, in mutant SOD1 astrocytes, there was an overall decrease in protein secretion with an increase in protein secretion through exosomes. There was an increase in mutant SOD1 secretion, but incorporation of the mutant protein into exosomes was reduced, as reported in motor neuron-like NSC-34 cells expressing G93A SOD1 (40).

**TABLE 2:** **Differentially secreted proteins in primary astrocytes derived from G93A SOD1 mice** Proteins were identified by MALDI-TOF mass spectrometry as reported under “Experimental Procedures” and in supplemental Table S1.

Spot*^a^*	Uniprot*^b^*	Protein name	Over*^c^*	Under*^d^*
			*-fold*	*-fold*
1	Q01853	Valosin-containing protein (VCP)/p97	1.9*^e^*	
2	O08553	DRP-2	2.9*^e^*	
3	P27773	Protein-disulfide isomerase A3 (ERp57)	2.1*^e^*	
4	P07214	Secreted protein acidic and rich in cysteine (SPARC)	3.7	
5	Q9R0P9	Ubiquitin carboxyl-terminal hydrolase isozyme L1	1.7	
6	P00441	Human SOD1	2.6*^e^*	

*^a^* Spot numbers (see Fig. 3*B*).

*^b^* Entry from the Uniprot Knowledgebase database.

*^c^* Proteins more present in G93A SOD1 astrocyte-conditioned media; values are volume -fold changes (G93A/WT).

*^d^* Proteins less present in G93A SOD1 astrocyte conditioned media; values are volume -fold changes (WT/G93A).

*^e^* Proteins identified in exosomes (ExoCarta database) (23).

##### Astrocyte-derived Exosomes Transfer SOD1 to Spinal Neurons

**FIGURE 5.**
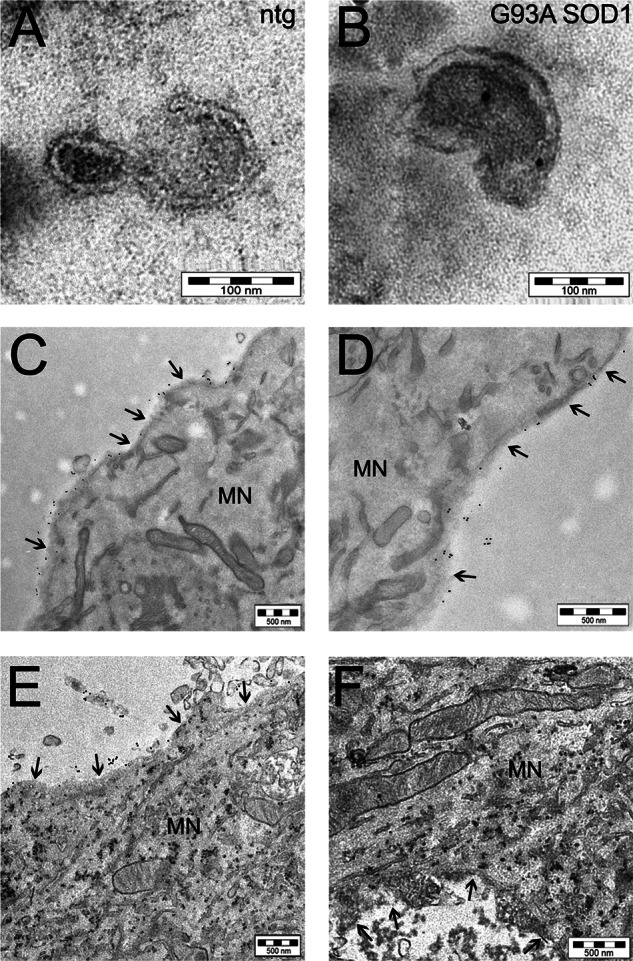
**Mutant SOD1 is transferred to spinal neurons through astrocyte-derived exosomes.** Electron microscopy pictures of exosomes with characteristic cup-shaped morphology from non-transgenic (*ntg*) (*A*) and G93A SOD1-expressing (*B*) astrocytes (*scale bar*, 100 nm). Immunoelectron microscopy using anti-human SOD1 antibody indicated SOD1 exclusively inside exosomes isolated from transgenic human SOD1-overexpressing astrocytes (in this case G93A SOD1; see also supplemental Fig. S1*A* for WT SOD1). *C* and *D*, two representative images at different magnification (*scale bar*, 500 nm) of cultured non-transgenic spinal neurons exposed to G93A SOD1-containing exosome-depleted fractions, showing the 10-nm electron-dense round gold particles labeling human SOD1 only outside the cell; treatment with G93A SOD1-containing exosomes (*E* and *F*) caused the diffusion inside the cytoplasm of the majority of human SOD1 (*scale bar*, 500 nm). The same results were obtained with WT SOD1-containing exosomes (supplemental Fig. S1*B*). The *arrows* indicate the plasma membrane of a motor neuron (*MN*).

Mutant SOD1 was associated with exosomes in astrocyte-conditioned medium. Exosomes have been extensively discussed as potential carriers in the dissemination of disease in neurodegenerative disorders (43). We therefore tested whether astrocyte-derived exosomes are a means of spreading SOD1-dependent toxicity. We isolated exosomal fractions from non-transgenic and transgenic SOD1-expressing astrocytes, WT and G93A, and analyzed them by electron microscopy after immunogold labeling for human SOD1. In this experimental setting, the anti-human SOD1 antibody can reveal only the overexpressed human SOD1 form, as shown in the representative images in Fig. 5, *A* and *B*, and supplemental Fig. S1*A*. We next treated non-transgenic spinal neuron cultures with exosome-depleted fractions or exosomes containing WT or G93A SOD1 pooling exosomal fractions from different astrocyte culture wells in order to treat spinal neurons with similar amounts of human SOD1 in all of the conditions. When exosome-depleted fractions were used for the treatments, SOD1 did not enter the cells. SOD1-immunogold-labeled particles were detectable only extracellularly (Fig. 5, *C* and *D*), with the SOD1 forms that remained anchored to the plasma membrane after medium removal before cell processing for electron microscopy. On the other hand, when exosomes were used for the treatments, G93A and WT SOD1 were readily transferred into spinal neurons, as demonstrated by the presence of diffused immunogold-labeled particles almost exclusively in the cytoplasm (Fig. 5, *E* and *F*, and supplemental Fig. S1*B*). Occasionally, there were groups of mutant SOD1-immunogold labeled particles, possibly indicating seeds of aggregation (Fig. 5, *E* and *F*). The specificity of the anti-human SOD1 antibody was further confirmed because non-transgenic spinal neurons treated with exosomes isolated from non-transgenic astrocytes did not show any immunogold-labeled particles (data not shown). Non-transgenic spinal neurons were also treated with non-processed conditioned medium from G93A SOD1-expressing astrocytes. In this case, only a few immunogold-labeled particles were inside the neurons (supplemental Fig. S2), in agreement with the fact that the medium contained only a limited number of exosomes. In conclusion, we found that astrocyte-derived exosomes are able to transfer SOD1 to spinal neurons. This may be a mechanism by which astrocytes contribute to the spreading of disease in ALS.

##### Astrocyte-derived Exosomes Containing Mutant SOD1 Induce Selective Motor Neuron Death

**FIGURE 6.**
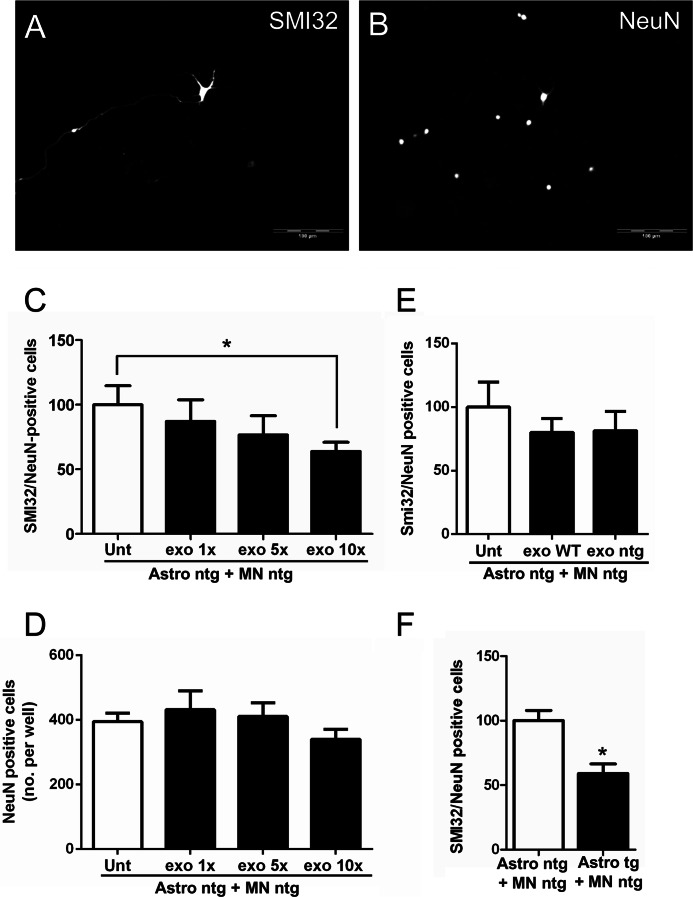
**G93A SOD1 astrocyte-derived exosomes are sufficient to induce motor neuron death.** In spinal neuron-astrocyte co-cultures, motor neuron survival was determined by the ratio of the number of SMI32-positive cells (*A*) to total NeuN-positive cells (*B*) in the same frame (20 frames/well). *Scale bar*, 100 μm. *C*, non-transgenic motor neurons plated on non-transgenic astrocytes (*Astro ntg* + *MN ntg*) survived significantly less after 6 days in culture when treated with increasing concentrations (1×, 5×, and 10×) of G93A SOD1 astrocyte-derived exosomes (post-test for linear trend *p* = 0.03; *n* = 8 for each condition). The 10× exosome concentration (*exo 10*×) significantly reduced the survival of motor neurons compared with untreated (*Unt*) (Student's *t* test, *p* = 0.01). *D*, neurons (NeuN-positive cells) did not show a significantly reduced survival, compared with untreated, by post-test linear trend (*p* = 0.27), even with the 10× exosome concentration (Student's *t* test, *p* > 0.05). Values are means of NeuN-positive cells per well. *E*, non-transgenic motor neurons plated on non-transgenic astrocytes showed no significantly reduced survival after 6 days in culture when treated with a 10× concentration of WT SOD1 (*exo WT*) or non-transgenic (*exo ntg*) astrocyte-derived exosomes with respect to untreated cells, Student's *t* test (*n* = 8 for each condition). *F*, a toxic effect was observed when non-transgenic spinal neurons were plated on G93A SOD1 astrocytes (*Astro tg* + *MN ntg*) after 6 days in culture (Student's *t* test, *p* = 0.0003; *n* = 17 for each condition). *Error bars*, S.E.

Previous studies have shown that astrocytes from mutant SOD1 mouse models can cause motor neuron death *in vitro* by means of secreted factors (5–10), but the exact nature of these factors is not known. We investigated whether isolated mutant SOD1 astrocyte-derived exosomes were sufficient to induce motor neuron death *in vitro*, treating non-transgenic spinal neuron-astrocyte co-cultures with increasing concentrations of exosome preparations from mutant SOD1 astrocytes. After 6 days in culture, we measured motor neuron viability as the ratio between the number of SMI32-postive cells, motor neurons, and the number of NeuN-positive cells, all neuronal cells (Fig. 6, *A* and *B*). Fig. 6*C* shows how motor neuron viability decreased with increased concentrations of the exosome preparation, which instead did not affect neuron viability (Fig. 6*D*). Although the highest concentration of exosomes from non-transgenic or WT SOD1 astrocytes had no significant effect on motor neuron (Fig. 6*E*), G93A SOD1-containing exosomes significantly reduced motor neuron viability (36%). A similar effect was observed in spinal neuron-astrocyte co-cultures, where non-transgenic spinal neurons were plated on G93A SOD1 astrocytes and maintained in culture for 6 days (Fig. 6*F*). In our assay, non-transgenic co-cultures treated with unprocessed mutant SOD1 astrocyte-conditioned media showed no significant decrease in motor neuron viability (data not shown). This indicates that to induce selective motor neuron death, it is necessary to have either a large number of mutant SOD1-containing exosomes or direct contact between astrocytes and motor neurons to allow continuous delivery of toxic exosomes into cells, as in the case of non-transgenic spinal neurons plated on G93A SOD1 astrocytes.

## DISCUSSION

*In vivo* studies using mutant-SOD1 mouse models have demonstrated that astrocytes have a role in the progression (13) and more recently also in the onset of ALS (14). The *in vivo* toxicity of mutant SOD1-expressing astrocytes toward motor neurons has also been confirmed by transplantation of mutant SOD1 astrocyte progenitor cells in WT mice (44), in which graft-derived mutant SOD1 astrocytes induced motor neuron death and associated pathological changes. Considering the *in vivo* findings together with the evidence *in vitro* (5–10), it is now clear that toxic factor(s) produced and released by astrocytes mediate motor neuron death in mutant SOD1-linked experimental models. We investigated the effect of mutant SOD1 expression on the proteome and secretome of these specific cells using a proteomic approach based on poststained two-dimensional gel electrophoresis. We observed dysregulation of proteins in secretory pathways, ER proteins (HSP90B1, GRP78, and protein-disulfide isomerase), and proteins involved in vesicle trafficking (RabGDI and RhoGDI) (35, 36). Alterations of these proteins have often been linked to disturbances in the secretory apparatus and ER stress in misfolded protein-associated diseases, including ALS (45–48). However, whereas in neuronal cells these proteins are usually up-regulated and associated with aggregation, in our mutant SOD1-expressing astrocytes they are down-regulated, indicating the activation of a different mechanism and possible impairment of protein secretion. Indeed, together with this alteration there was a substantial decrease in secreted proteins. However, we found an up-regulation of exosome release and an increase in the level of exosome-associated proteins in the conditioned medium of G93A SOD1 astrocytes. This might explain the results of the proteomic analysis of astrocytes, where, with mutant SOD1 expression, there were decreased levels of proteins such as CypA, RhoGDI, pyruvate kinase, α-enolase, and 14-3-3 protein, which are the most common components of canonical exosomes (42).

Not much is known about exosomes of astrocyte origin. Interestingly, astrocyte-derived exosomes have been recently reported in rat CSF (49), implying that our findings may be relevant *in vivo* too. Many different functions have been attributed to exosomes, depending on their origin. For some cells, exosome secretion is a way to dispose of unwanted proteins, sending them to a drainage system. Besides SOD1, cytosolic misfolded proteins involved in neurodegenerative diseases, such as α-synuclein and Tau, have been found in exosomes (26), and a high level of exosome-associated phosphorylated Tau was found in CSF of Alzheimer patients (50). It is possible that brain cells, including astrocytes, use exosome release as a means of clearance for misfolded and potentially toxic proteins. However, exosome release can have detrimental effects on neighboring cells (51, 52) and is considered a potential means for disease spreading in neurodegenerative disorders (26).

Secretome analysis indicated that conditioned media from G93A SOD1 astrocytes were enriched in a selected number of proteins, including mutant SOD1. In contrast, previous studies have detected low levels of mutant SOD1 in conditioned media of motor neuron-like NSC-34 cells expressing a variety of SOD1 mutants (16). Impaired secretion of mutant SOD1 was associated with formation of inclusions and toxicity, which were attenuated by extracellular targeting of mutant SOD1. The differences in behavior may reflect the peculiarities of the different cell types. Astrocytes, in the presence of toxic mutant SOD1, may activate specific conventional and unconventional secretory pathways as a protective mechanism. Our study confirmed that SOD1 is also secreted through exosomes, in agreement with previous findings (16, 40). As in NSC-34 cell exosomes (40), mutant SOD1 is less present than WT SOD1 in astrocyte-derived exosomes. Probably, although astrocytes produce more exosomes to eliminate G93A SOD1, the mutation disfavors its incorporation, maybe because of intracellular entrapment of the misfolded mutant protein or its aberrant interaction with chaperone-like proteins, such as chromogranins, in neurons that could mediate its secretion through the ER-Golgi network (17). It is hardly surprising that we did not detect chromogranins in our proteomic analysis because these proteins are abundantly expressed in neurons, but their expression is very low in astrocytes (17). Therefore, other protein factors, yet to be identified, may be involved in mutant SOD1 exosome-independent secretion in astrocytes, which is apparently the main secretory pathway.

VCP/p97 was also enriched in the conditioned media of G93A SOD1 astrocytes. VCP/p97, a member of the AAA-ATPase superfamily, is involved in different ways in protein degradation (53, 54). It has also been associated with various neurodegenerative diseases and is found in aggregates isolated from patients. In a previous study, we found that VCP/p97 (referred to as transitional endoplasmic reticulum ATPase) was recovered in mutant SOD1-rich aggregates from the spinal cord in the G93A SOD1 mouse model, already at a presymptomatic stage of the disease (28). Possibly, VCP/p97, which interacts directly or indirectly with misfolded mutant SOD1 (55, 56), is sorted with it into exosomes as unwanted material to be shuttled out of astrocytes. VCP/p97 mutations have been associated with familial and sporadic ALS cases (57, 58). It would be interesting now to investigate the behavior of VCP/p97 mutants in secretion and the possible implications in pathogenesis.

We can therefore hypothesize that astrocytes, unlike motor neurons (16), activate secretory pathways that can selectively eliminate mutant SOD1 and possibly other misfolded or oxidized proteins that may cause intracellular toxicity. This could explain the ability of astrocytes to limit the formation of intracellular aggregates and overcome their toxicity although, on the other hand, ultimately to exert a toxic effect on neighboring motor neurons through extracellular release. Therapeutic interventions aimed at the extracellular pool of mutant/misfolded SOD1 have been successful in delaying mortality in G93A SOD1 mice (59–61). We cannot exclude the possibility that other proteins/molecules are involved in toxicity, and targeting these extracellularly too might improve the therapeutic effect.

It remains to be established how extracellular mutant SOD1, free and associated with exosomes, affects motor neurons. Studies *in vitro* have shown that free extracellular mutant SOD1 can induce motor neuron injury by activation of microglia, increasing their release of proinflammatory cytokines and free radicals, through Toll-like receptor and CD14 pathways (17, 62). Moreover, exogenously applied mutant SOD1 aggregates enter Neuro-2a cells, where they can seed aggregation of the normal cytoplasmic mutant SOD1 (63). Here we showed for the first time that mutant SOD1 primary astrocyte cultures are secreting mutant SOD1-containing exosomes, which are able to transfer mutant SOD1 into spinal neuron cultures, and are sufficient to induce selective motor neuron death. Exosomes from WT SOD1 astrocytes are also able to transfer human SOD1 into cells but have no toxic effect. We cannot exclude the possibility that other factors besides mutant SOD1 may contribute to toxicity. However, because the increased protein concentration is thought to be the main determinant for protein aggregation, one could envisage that *in vivo* continuous delivery of mutant SOD1 by astrocytes to neighboring motor neurons might facilitate aggregation and disease spreading. This is in agreement with a model in which motor neuron degeneration in ALS is a focal process that actively propagates in a orderly fashion to adjacent regions (64). Evidence for the spreading of SOD1-dependent toxicity as a general feature of human ALS has been proposed recently (65), indicating that this exosome-dependent transfer may be important also in sporadic cases. In view of this idea, a novel therapeutic approach should consider targeting exosomes as one way to contain the progression of the disease.

**FIGURE 7.**
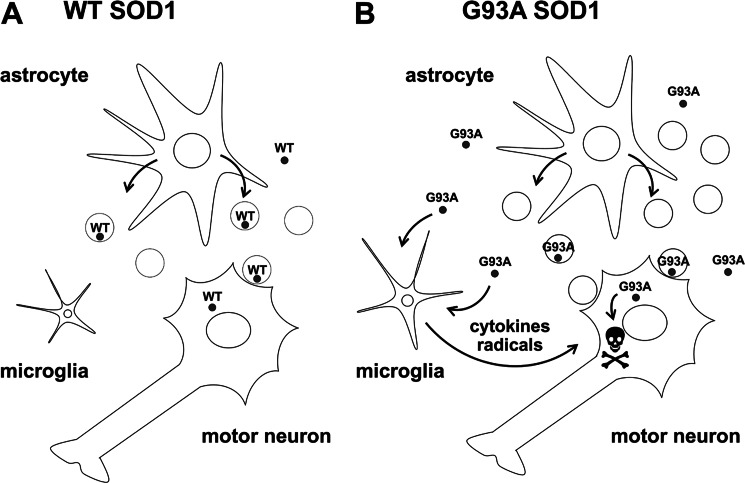
**Proposed pathogenic mechanism for G93A SOD1-expressing astrocytes.** *A*, astrocytes normally release WT SOD1 in the extracellular space through exosomal and non-exosomal secretory pathways. *B*, astrocytes expressing mutant SOD1 (G93A SOD1) activate unconventional secretory pathways, possibly to protect themselves from mutant SOD1-associated toxicity and release more exosomes. Exosomes contribute to the spreading of the disease by continuous transfer of toxic molecules, including G93A SOD1, to neighboring motor neurons. G93A SOD1 secreted by astrocytes contributes to motor neuron injury indirectly, increasing the release of proinflammatory cytokines and free radicals by activated microglia (17, 62) and directly, once it is transferred to inside motor neurons by exosomes, by facilitating the initiation of aggregation.

We conclude that mutant SOD1 can alter secretory pathways and exosome release in primary astrocyte cultures. We suggest that mutant SOD1 astrocytes may ultimately exert a toxic effect on neighboring motor neurons, indirectly by microglial-mediated injury (17, 62) and directly by transferring toxic exosomes (Fig. 7).

## Supplementary Material

Supplemental Data

## Footnotes

5 The abbreviations used are:

ALS: amyotrophic lateral sclerosis
CSF: cerebrospinal fluid
GFAP: glial fibrillary acidic protein
IPG: immobilized pH gradient
WB: Western blotting
ER: endoplasmic reticulum
VCP: valosin-containing protein.

## References

[1] Ilieva H. Polymenidou M. Cleveland D. W. (2009) Non-cell autonomous toxicity in neurodegenerative disorders. ALS and beyond. J. Cell Biol. 187, 761–772 19951898 10.1083/jcb.200908164 PMC2806318

[2] Howland D. S. Liu J. She Y. Goad B. Maragakis N. J. Kim B. Erickson J. Kulik J. DeVito L. Psaltis G. DeGennaro L. J. Cleveland D. W. Rothstein J. D. (2002) Focal loss of the glutamate transporter EAAT2 in a transgenic rat model of SOD1 mutant-mediated amyotrophic lateral sclerosis (ALS). Proc. Natl. Acad. Sci. U.S.A. 99, 1604–1609 11818550 10.1073/pnas.032539299 PMC122237

[3] Rothstein J. D. Van Kammen M. Levey A. I. Martin L. J. Kuncl R. W. (1995) Selective loss of glial glutamate transporter GLT-1 in amyotrophic lateral sclerosis. Ann. Neurol. 38, 73–84 7611729 10.1002/ana.410380114

[4] Bendotti C. Tortarolo M. Suchak S. K. Calvaresi N. Carvelli L. Bastone A. Rizzi M. Rattray M. Mennini T. (2001) Transgenic SOD1 G93A mice develop reduced GLT-1 in spinal cord without alterations in cerebrospinal fluid glutamate levels. J. Neurochem. 79, 737–746 11723166 10.1046/j.1471-4159.2001.00572.x

[5] Aebischer J. Cassina P. Otsmane B. Moumen A. Seilhean D. Meininger V. Barbeito L. Pettmann B. Raoul C. (2011) IFNγ triggers a LIGHT-dependent selective death of motoneurons contributing to the non-cell-autonomous effects of mutant SOD1. Cell Death Differ. 18, 754–768 21072055 10.1038/cdd.2010.143 PMC3131923

[6] Díaz-Amarilla P. Olivera-Bravo S. Trias E. Cragnolini A. Martínez-Palma L. Cassina P. Beckman J. Barbeito L. (2011) Phenotypically aberrant astrocytes that promote motoneuron damage in a model of inherited amyotrophic lateral sclerosis. Proc. Natl. Acad. Sci. U.S.A. 108, 18126–18131 22010221 10.1073/pnas.1110689108 PMC3207668

[7] Marchetto M. C. Muotri A. R. Mu Y. Smith A. M. Cezar G. G. Gage F. H. (2008) Non-cell-autonomous effect of human SOD1 G37R astrocytes on motor neurons derived from human embryonic stem cells. Cell Stem Cell 3, 649–657 19041781 10.1016/j.stem.2008.10.001

[8] Di Giorgio F. P. Carrasco M. A. Siao M. C. Maniatis T. Eggan K. (2007) Non-cell autonomous effect of glia on motor neurons in an embryonic stem cell-based ALS model. Nat. Neurosci. 10, 608–614 17435754 10.1038/nn1885 PMC3139463

[9] Nagai M. Re D. B. Nagata T. Chalazonitis A. Jessell T. M. Wichterle H. Przedborski S. (2007) Astrocytes expressing ALS-linked mutated SOD1 release factors selectively toxic to motor neurons. Nat. Neurosci. 10, 615–622 17435755 10.1038/nn1876 PMC3799799

[10] Vargas M. R. Pehar M. Cassina P. Beckman J. S. Barbeito L. (2006) Increased glutathione biosynthesis by Nrf2 activation in astrocytes prevents p75NTR-dependent motor neuron apoptosis. J. Neurochem. 97, 687–696 16524372 10.1111/j.1471-4159.2006.03742.x

[11] Lepore A. C. Rauck B. Dejea C. Pardo A. C. Rao M. S. Rothstein J. D. Maragakis N. J. (2008) Focal transplantation-based astrocyte replacement is neuroprotective in a model of motor neuron disease. Nat. Neurosci. 11, 1294–1301 18931666 10.1038/nn.2210 PMC2656686

[12] Vargas M. R. Johnson D. A. Sirkis D. W. Messing A. Johnson J. A. (2008) Nrf2 activation in astrocytes protects against neurodegeneration in mouse models of familial amyotrophic lateral sclerosis. J. Neurosci. 28, 13574–13581 19074031 10.1523/JNEUROSCI.4099-08.2008 PMC2866507

[13] Yamanaka K. Chun S. J. Boillee S. Fujimori-Tonou N. Yamashita H. Gutmann D. H. Takahashi R. Misawa H. Cleveland D. W. (2008) Astrocytes as determinants of disease progression in inherited amyotrophic lateral sclerosis. Nat. Neurosci. 11, 251–253 18246065 10.1038/nn2047 PMC3137510

[14] Wang L. Gutmann D. H. Roos R. P. (2011) Astrocyte loss of mutant SOD1 delays ALS disease onset and progression in G85R transgenic mice. Hum. Mol. Genet. 20, 286–293 20962037 10.1093/hmg/ddq463

[15] Lafon-Cazal M. Adjali O. Galéotti N. Poncet J. Jouin P. Homburger V. Bockaert J. Marin P. (2003) Proteomic analysis of astrocytic secretion in the mouse. Comparison with the cerebrospinal fluid proteome. J. Biol. Chem. 278, 24438–24448 12709418 10.1074/jbc.M211980200

[16] Turner B. J. Atkin J. D. Farg M. A. Zang D. W. Rembach A. Lopes E. C. Patch J. D. Hill A. F. Cheema S. S. (2005) Impaired extracellular secretion of mutant superoxide dismutase 1 associates with neurotoxicity in familial amyotrophic lateral sclerosis. J. Neurosci. 25, 108–117 15634772 10.1523/JNEUROSCI.4253-04.2005 PMC6725218

[17] Urushitani M. Sik A. Sakurai T. Nukina N. Takahashi R. Julien J. P. (2006) Chromogranin-mediated secretion of mutant superoxide dismutase proteins linked to amyotrophic lateral sclerosis. Nat. Neurosci. 9, 108–118 16369483 10.1038/nn1603

[18] Jacobsson J. Jonsson P. A. Andersen P. M. Forsgren L. Marklund S. L. (2001) Superoxide dismutase in CSF from amyotrophic lateral sclerosis patients with and without CuZn-superoxide dismutase mutations. Brain 124, 1461–1466 11408340 10.1093/brain/124.7.1461

[19] Zetterström P. Andersen P. M. Brännström T. Marklund S. L. (2011) Misfolded superoxide dismutase-1 in CSF from amyotrophic lateral sclerosis patients. J Neurochem 117, 91–99 21226712 10.1111/j.1471-4159.2011.07177.x

[20] Delcourt N. Jouin P. Poncet J. Demey E. Mauger E. Bockaert J. Marin P. Galéotti N. (2005) Difference in mass analysis using labeled lysines (DIMAL-K). A new, efficient proteomic quantification method applied to the analysis of astrocytic secretomes. Mol. Cell. Proteomics 4, 1085–1094 15905179 10.1074/mcp.M500040-MCP200

[21] Greco T. M. Seeholzer S. H. Mak A. Spruce L. Ischiropoulos H. (2010) Quantitative mass spectrometry-based proteomics reveals the dynamic range of primary mouse astrocyte protein secretion. J. Proteome Res. 9, 2764–2774 20329800 10.1021/pr100134n PMC2866110

[22] Keene S. D. Greco T. M. Parastatidis I. Lee S. H. Hughes E. G. Balice-Gordon R. J. Speicher D. W. Ischiropoulos H. (2009) Mass spectrometric and computational analysis of cytokine-induced alterations in the astrocyte secretome. Proteomics 9, 768–782 19132682 10.1002/pmic.200800385 PMC2667946

[23] Mathivanan S. Simpson R. J. (2009) ExoCarta. A compendium of exosomal proteins and RNA. Proteomics 9, 4997–5000 19810033 10.1002/pmic.200900351

[24] Février B. Raposo G. (2004) Exosomes. Endosomal-derived vesicles shipping extracellular messages. Curr. Opin. Cell Biol. 16, 415–421 15261674 10.1016/j.ceb.2004.06.003

[25] Valadi H. Ekström K. Bossios A. Sjöstrand M. Lee J. J. Lötvall J. O. (2007) Exosome-mediated transfer of mRNAs and microRNAs is a novel mechanism of genetic exchange between cells. Nat. Cell Biol. 9, 654–659 17486113 10.1038/ncb1596

[26] Bellingham S. A. Guo B. B. Coleman B. M. Hill A. F. (2012) Exosomes. Vehicles for the transfer of toxic proteins associated with neurodegenerative diseases? Front. Physiol. 3, 124 22563321 10.3389/fphys.2012.00124 PMC3342525

[27] Tortarolo M. Crossthwaite A. J. Conforti L. Spencer J. P. Williams R. J. Bendotti C. Rattray M. (2004) Expression of SOD1 G93A or wild-type SOD1 in primary cultures of astrocytes down-regulates the glutamate transporter GLT-1. Lack of involvement of oxidative stress. J. Neurochem. 88, 481–493 14690536 10.1046/j.1471-4159.2003.02208.x

[28] Basso M. Samengo G. Nardo G. Massignan T. D'Alessandro G. Tartari S. Cantoni L. Marino M. Cheroni C. De Biasi S. Giordana M. T. Strong M. J. Estevez A. G. Salmona M. Bendotti C. Bonetto V. (2009) Characterization of detergent-insoluble proteins in ALS indicates a causal link between nitrative stress and aggregation in pathogenesis. PLoS One 4, e8130 19956584 10.1371/journal.pone.0008130 PMC2780298

[29] Casoni F. Basso M. Massignan T. Gianazza E. Cheroni C. Salmona M. Bendotti C. Bonetto V. (2005) Protein nitration in a mouse model of familial amyotrophic lateral sclerosis. Possible multifunctional role in the pathogenesis. J. Biol. Chem. 280, 16295–16304 15699043 10.1074/jbc.M413111200

[30] Biasini E. Massignan T. Fioriti L. Rossi V. Dossena S. Salmona M. Forloni G. Bonetto V. Chiesa R. (2006) Analysis of the cerebellar proteome in a transgenic mouse model of inherited prion disease reveals preclinical alteration of calcineurin activity. Proteomics 6, 2823–2834 16572473 10.1002/pmic.200500620

[31] Pappin D. J. Hojrup P. Bleasby A. J. (1993) Rapid identification of proteins by peptide-mass fingerprinting. Curr. Biol. 3, 327–332 15335725 10.1016/0960-9822(93)90195-t

[32] Peviani M. Kurosaki M. Terao M. Lidonnici D. Gensano F. Battaglia E. Tortarolo M. Piva R. Bendotti C. (2012) Lentiviral vectors carrying enhancer elements of Hb9 promoter drive selective transgene expression in mouse spinal cord motor neurons. J. Neurosci. Methods 205, 139–147 22245491 10.1016/j.jneumeth.2011.12.024

[33] Migheli A. Cordera S. Bendotti C. Atzori C. Piva R. Schiffer D. (1999) S-100β protein is upregulated in astrocytes and motor neurons in the spinal cord of patients with amyotrophic lateral sclerosis. Neurosci. Lett. 261, 25–28 10081918 10.1016/s0304-3940(98)01001-5

[34] Fujita K. Yamauchi M. Matsui T. Titani K. Takahashi H. Kato T. Isomura G. Ando M. Nagata Y. (1998) Increase of glial fibrillary acidic protein fragments in the spinal cord of motor neuron degeneration mutant mouse. Brain Res. 785, 31–40 9526038 10.1016/s0006-8993(97)00612-4

[35] Ridley A. J. (2006) Rho GTPases and actin dynamics in membrane protrusions and vesicle trafficking. Trends Cell Biol. 16, 522–529 16949823 10.1016/j.tcb.2006.08.006

[36] Stenmark H. (2009) Rab GTPases as coordinators of vesicle traffic. Nat. Rev. Mol. Cell Biol. 10, 513–525 19603039 10.1038/nrm2728

[37] Potokar M. Kreft M. Li L. Daniel Andersson J. Pangrsic T. Chowdhury H. H. Pekny M. Zorec R. (2007) Cytoskeleton and vesicle mobility in astrocytes. Traffic 8, 12–20 17229312 10.1111/j.1600-0854.2006.00509.x

[38] Fountoulakis M. Juranville J. F. Manneberg M. (1992) Comparison of the Coomassie Brilliant Blue, bicinchoninic acid and Lowry quantitation assays, using non-glycosylated and glycosylated proteins. J. Biochem. Biophys. Methods 24, 265–274 1640058 10.1016/0165-022x(94)90078-7

[39] Petersen T. N. Brunak S. von Heijne G. Nielsen H. (2011) SignalP 4.0. Discriminating signal peptides from transmembrane regions. Nat. Methods 8, 785–786 21959131 10.1038/nmeth.1701

[40] Gomes C. Keller S. Altevogt P. Costa J. (2007) Evidence for secretion of Cu Zn superoxide dismutase via exosomes from a cell model of amyotrophic lateral sclerosis. Neurosci. Lett. 428, 43–46 17942226 10.1016/j.neulet.2007.09.024

[41] Fevrier B. Vilette D. Archer F. Loew D. Faigle W. Vidal M. Laude H. Raposo G. (2004) Cells release prions in association with exosomes. Proc. Natl. Acad. Sci. U.S.A. 101, 9683–9688 15210972 10.1073/pnas.0308413101 PMC470735

[42] Théry C. Ostrowski M. Segura E. (2009) Membrane vesicles as conveyors of immune responses. Nat. Rev. Immunol. 9, 581–593 19498381 10.1038/nri2567

[43] Schneider A. Simons M. (2013) Exosomes. Vesicular carriers for intercellular communication in neurodegenerative disorders. Cell Tissue Res. 352, 33–47 22610588 10.1007/s00441-012-1428-2 PMC3602607

[44] Papadeas S. T. Kraig S. E. O'Banion C. Lepore A. C. Maragakis N. J. (2011) Astrocytes carrying the superoxide dismutase 1 (SOD1G93A) mutation induce wild-type motor neuron degeneration *in vivo*. Proc. Natl. Acad. Sci. U.S.A. 108, 17803–17808 21969586 10.1073/pnas.1103141108 PMC3203804

[45] Kaufman R. J. (1999) Stress signaling from the lumen of the endoplasmic reticulum. Coordination of gene transcriptional and translational controls. Genes Dev. 13, 1211–1233 10346810 10.1101/gad.13.10.1211

[46] Massignan T. Biasini E. Lauranzano E. Veglianese P. Pignataro M. Fioriti L. Harris D. A. Salmona M. Chiesa R. Bonetto V. (2010) Mutant prion protein expression is associated with an alteration of the Rab GDP dissociation inhibitor α (GDI)/Rab11 pathway. Mol Cell Proteomics 9, 611–622 19996123 10.1074/mcp.M900271-MCP200 PMC2860234

[47] Atkin J. D. Farg M. A. Turner B. J. Tomas D. Lysaght J. A. Nunan J. Rembach A. Nagley P. Beart P. M. Cheema S. S. Horne M. K. (2006) Induction of the unfolded protein response in familial amyotrophic lateral sclerosis and association of protein-disulfide isomerase with superoxide dismutase 1. J. Biol. Chem. 281, 30152–30165 16847061 10.1074/jbc.M603393200

[48] Atkin J. D. Farg M. A. Walker A. K. McLean C. Tomas D. Horne M. K. (2008) Endoplasmic reticulum stress and induction of the unfolded protein response in human sporadic amyotrophic lateral sclerosis. Neurobiol. Dis. 30, 400–407 18440237 10.1016/j.nbd.2008.02.009

[49] Verderio C. Muzio L. Turola E. Bergami A. Novellino L. Ruffini F. Riganti L. Corradini I. Francolini M. Garzetti L. Maiorino C. Servida F. Vercelli A. Rocca M. Dalla Libera D. Martinelli V. Comi G. Martino G. Matteoli M. Furlan R. (2012) Myeloid microvesicles are a marker and therapeutic target for neuroinflammation. Ann. Neurol. 72, 610–624 23109155 10.1002/ana.23627

[50] Saman S. Kim W. Raya M. Visnick Y. Miro S. Saman S. Jackson B. McKee A. C. Alvarez V. E. Lee N. C. Hall G. F. (2012) Exosome-associated Tau is secreted in tauopathy models and is selectively phosphorylated in cerebrospinal fluid in early Alzheimer disease. J. Biol. Chem. 287, 3842–3849 22057275 10.1074/jbc.M111.277061 PMC3281682

[51] Emmanouilidou E. Melachroinou K. Roumeliotis T. Garbis S. D. Ntzouni M. Margaritis L. H. Stefanis L. Vekrellis K. (2010) Cell-produced α-synuclein is secreted in a calcium-dependent manner by exosomes and impacts neuronal survival. J. Neurosci. 30, 6838–6851 20484626 10.1523/JNEUROSCI.5699-09.2010 PMC3842464

[52] Wang G. Dinkins M. He Q. Zhu G. Poirier C. Campbell A. Mayer-Proschel M. Bieberich E. (2012) Astrocytes secrete exosomes enriched with proapoptotic ceramide and prostate apoptosis response 4 (PAR-4). Potential mechanism of apoptosis induction in Alzheimer disease (AD). J. Biol. Chem. 287, 21384–21395 22532571 10.1074/jbc.M112.340513 PMC3375560

[53] Meyer H. Bug M. Bremer S. (2012) Emerging functions of the VCP/p97 AAA-ATPase in the ubiquitin system. Nat. Cell Biol. 14, 117–123 22298039 10.1038/ncb2407

[54] Piccirillo R. Goldberg A. L. (2012) The p97/VCP ATPase is critical in muscle atrophy and the accelerated degradation of muscle proteins. EMBO J. 31, 3334–3350 22773186 10.1038/emboj.2012.178 PMC3411080

[55] Choi J. S. Lee D. H. (2010) CHIP promotes the degradation of mutant SOD1 by reducing its interaction with VCP and S6/S6′ subunits of 26S proteasome. Anim. Cells Syst. 14, 1–10

[56] Nishitoh H. Kadowaki H. Nagai A. Maruyama T. Yokota T. Fukutomi H. Noguchi T. Matsuzawa A. Takeda K. Ichijo H. (2008) ALS-linked mutant SOD1 induces ER stress- and ASK1-dependent motor neuron death by targeting Derlin-1. Genes Dev. 22, 1451–1464 18519638 10.1101/gad.1640108 PMC2418582

[57] Abramzon Y. Johnson J. O. Scholz S. W. Taylor J. P. Brunetti M. Calvo A. Mandrioli J. Benatar M. Mora G. Restagno G. Chio A. Traynor B. J. (2012) Valosin-containing protein (VCP) mutations in sporadic amyotrophic lateral sclerosis. Neurobiol. Aging 33, 2231.e1–2231.e6 22572540 10.1016/j.neurobiolaging.2012.04.005 PMC3391327

[58] Johnson J. O. Mandrioli J. Benatar M. Abramzon Y. Van Deerlin V. M. Trojanowski J. Q. Gibbs J. R. Brunetti M. Gronka S. Wuu J. Ding J. McCluskey L. Martinez-Lage M. Falcone D. Hernandez D. G. Arepalli S. Chong S. Schymick J. C. Rothstein J. Landi F. Wang Y. D. Calvo A. Mora G. Sabatelli M. Monsurrò M. R. Battistini S. Salvi F. Spataro R. Sola P. Borghero G., ITALSGEN Consortium, Galassi G. Scholz S. W. Taylor J. P. Restagno G. Chiò A. Traynor B. J. (2010) Exome sequencing reveals VCP mutations as a cause of familial ALS. Neuron 68, 857–864 21145000 10.1016/j.neuron.2010.11.036 PMC3032425

[59] Gros-Louis F. Soucy G. Larivière R. Julien J. P. (2010) Intracerebroventricular infusion of monoclonal antibody or its derived Fab fragment against misfolded forms of SOD1 mutant delays mortality in a mouse model of ALS. J Neurochem 113, 1188–1199 20345765 10.1111/j.1471-4159.2010.06683.x

[60] Urushitani M. Ezzi S. A. Julien J. P. (2007) Therapeutic effects of immunization with mutant superoxide dismutase in mice models of amyotrophic lateral sclerosis. Proc. Natl. Acad. Sci. U.S.A. 104, 2495–2500 17277077 10.1073/pnas.0606201104 PMC1790867

[61] Liu H. N. Tjostheim S. Dasilva K. Taylor D. Zhao B. Rakhit R. Brown M. Chakrabartty A. McLaurin J. Robertson J. (2012) Targeting of monomer/misfolded SOD1 as a therapeutic strategy for amyotrophic lateral sclerosis. J. Neurosci. 32, 8791–8799 22745481 10.1523/JNEUROSCI.5053-11.2012 PMC6622344

[62] Zhao W. Beers D. R. Henkel J. S. Zhang W. Urushitani M. Julien J. P. Appel S. H. (2010) Extracellular mutant SOD1 induces microglial-mediated motoneuron injury. Glia 58, 231–243 19672969 10.1002/glia.20919 PMC2784168

[63] Münch C. O'Brien J. Bertolotti A. (2011) Prion-like propagation of mutant superoxide dismutase-1 misfolding in neuronal cells. Proc. Natl. Acad. Sci. U.S.A. 108, 3548–3553 21321227 10.1073/pnas.1017275108 PMC3048161

[64] Ravits J. M. La Spada A. R. (2009) ALS motor phenotype heterogeneity, focality, and spread. Deconstructing motor neuron degeneration. Neurology 73, 805–811 19738176 10.1212/WNL.0b013e3181b6bbbd PMC2739608

[65] Haidet-Phillips A. M. Hester M. E. Miranda C. J. Meyer K. Braun L. Frakes A. Song S. Likhite S. Murtha M. J. Foust K. D. Rao M. Eagle A. Kammesheidt A. Christensen A. Mendell J. R. Burghes A. H. Kaspar B. K. (2011) Astrocytes from familial and sporadic ALS patients are toxic to motor neurons. Nat. Biotechnol. 29, 824–828 21832997 10.1038/nbt.1957 PMC3170425

