# Supplementary material for: Mutant Copper-Zinc Superoxide Dismutase (SOD1) Induces Protein Secretion Pathway Alterations and Exosome Release in Astrocytes: IMPLICATIONS FOR DISEASE SPREADING AND MOTOR NEURON PATHOLOGY IN AMYOTROPHIC LATERAL SCLEROSIS
Source: J Biol Chem. 2013 Apr 16;288(22):15699–711. doi: 10.1074/jbc.M112.425066 (PMC3668729; doi:10.1074/jbc.M112.425066)
Supplement: Supplemental Data [file supp_288_22_15699__index.html]

Mutant copper-zinc superoxide dismutase (SOD1) induces protein secretion pathway alterations and exosome release in astrocytes: implications for disease spreading and motor neuron pathology in amyotrophic lateral sclerosis — Mutant Copper-Zinc Superoxide Dismutase (SOD1) Induces Protein Secretion Pathway Alterations and Exosome Release in Astrocytes — Astrocyte-derived Exosomes in ALS — Supplemental Data 

# Mutant Copper-Zinc Superoxide Dismutase (SOD1) Induces Protein Secretion Pathway Alterations and Exosome Release in Astrocytes

## Supplemental Data

**Files in this Data Supplement:**

- Supplemental Figure 1 (.doc, 4.0 MB) - Figure S1. WT SOD1 is transferred to spinal neurons through astrocyte-derived exosomes.
- Supplemental Figure 2 (.doc, 1.4 MB) - Figure S2. Immuno-electron microscopy in non-transgenic spinal neurons treated with non processed conditioned medium from G93A SOD1-expressing astrocytes
- Supplemental Table 1 (.doc, 76 KB) - Table S1. Identification of differentially expressed proteins by MALDI-TOF mass spectrometry in primary astrocytes derived from G93A and WT SOD1 mice and their secreted proteins.
