# Supplementary material for: Mutant Copper-Zinc Superoxide Dismutase (SOD1) Induces Protein Secretion Pathway Alterations and Exosome Release in Astrocytes: IMPLICATIONS FOR DISEASE SPREADING AND MOTOR NEURON PATHOLOGY IN AMYOTROPHIC LATERAL SCLEROSIS
Source: J Biol Chem. 2013 Apr 16;288(22):15699–711. doi: 10.1074/jbc.M112.425066 (PMC3668729; doi:10.1074/jbc.M112.425066)
Supplement: Supplemental Data [file supp_M112.425066_jbc.M112.425066-1.doc]

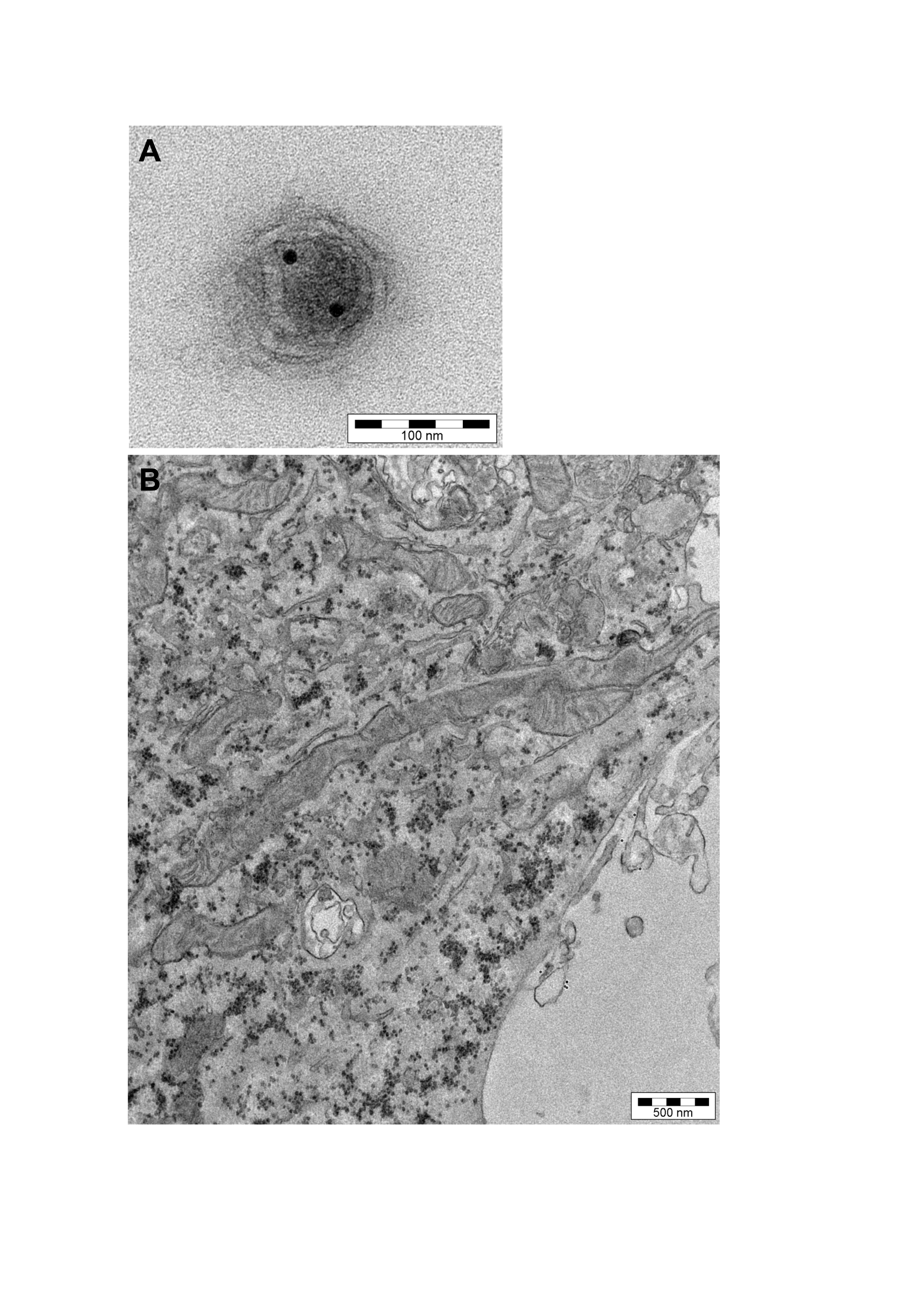


**Figure S1. WT SOD1 is transferred to spinal neurons through astrocyte-derived exosomes.** A, electron microscopy pictures of exosomes with characteristic cup-shaped morphology from WT SOD1 expressing astrocytes (scale bar 100 nm). Immuno-electron microscopy using anti-human SOD1 antibody indicated SOD1 exclusively inside exosomes isolated from transgenic human SOD1 over-expressing astrocytes (see also Figure 5A-B). B, a representative image of cultured non-transgenic spinal neurons exposed to WT SOD1-containing exosomes that shows the 10 nm electron-dense round gold particles labeling human SOD1 inside the cytoplasm of a motor neuron (scale bar 500 nm) (see also Figure 5). Arrows indicate the plasma membrane.
