# Supplementary material for: Mutant Copper-Zinc Superoxide Dismutase (SOD1) Induces Protein Secretion Pathway Alterations and Exosome Release in Astrocytes: IMPLICATIONS FOR DISEASE SPREADING AND MOTOR NEURON PATHOLOGY IN AMYOTROPHIC LATERAL SCLEROSIS
Source: J Biol Chem. 2013 Apr 16;288(22):15699–711. doi: 10.1074/jbc.M112.425066 (PMC3668729; doi:10.1074/jbc.M112.425066)
Supplement: Supplemental Data [file supp_M112.425066_jbc.M112.425066-2.doc]

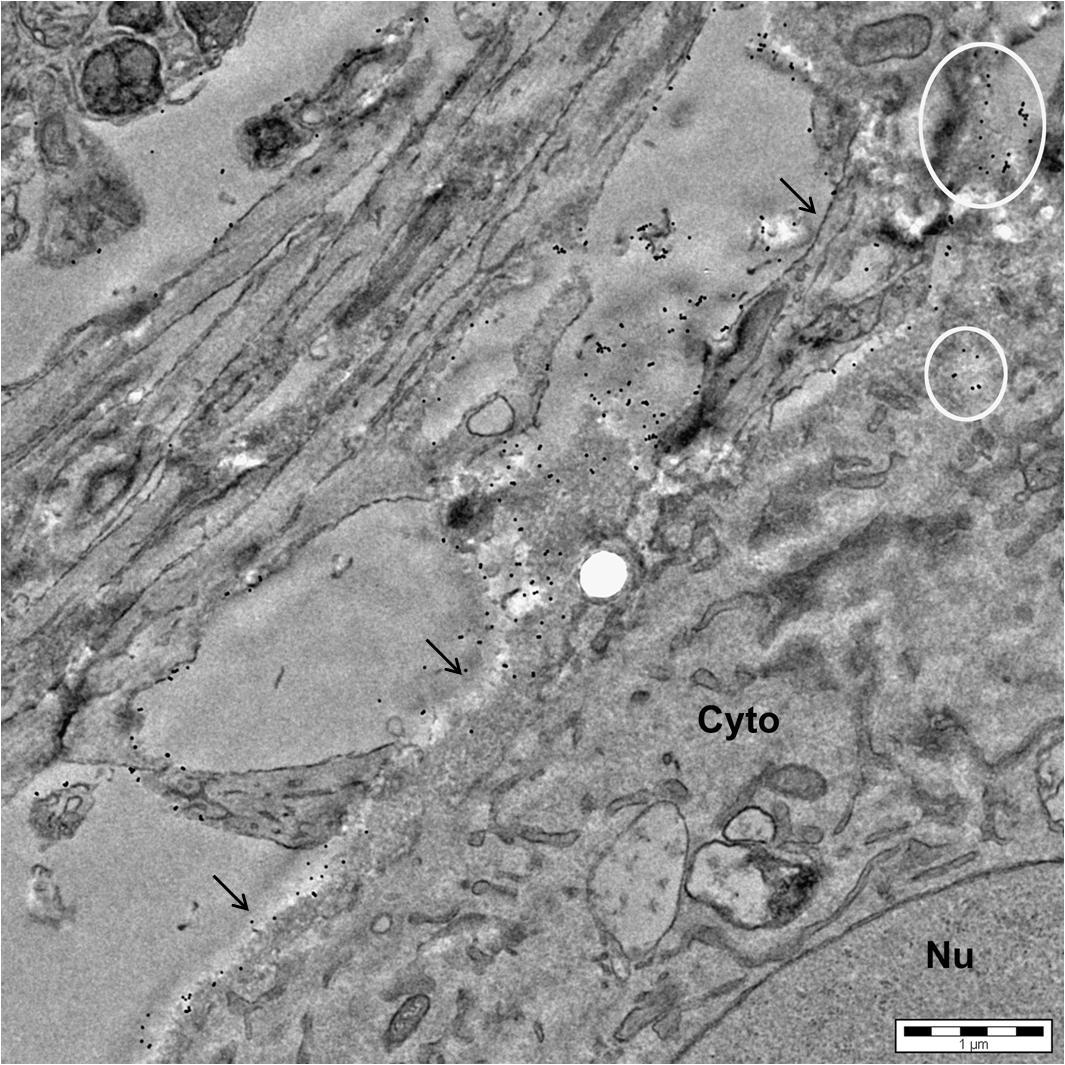


**Figure S2.** **Immuno-electron microscopy in non-transgenic spinal neurons treated with non processed conditioned medium from G93A SOD1-expressing astrocytes**. The image shows that there are only few particles inside the cytoplasm (Cyto) (white encircled in the figure). Arrows indicate the plasma membrane; Nu, nucleus; (Scale bar 1 μm).
