# Supplementary material for: Mutant Copper-Zinc Superoxide Dismutase (SOD1) Induces Protein Secretion Pathway Alterations and Exosome Release in Astrocytes: IMPLICATIONS FOR DISEASE SPREADING AND MOTOR NEURON PATHOLOGY IN AMYOTROPHIC LATERAL SCLEROSIS
Source: J Biol Chem. 2013 Apr 16;288(22):15699–711. doi: 10.1074/jbc.M112.425066 (PMC3668729; doi:10.1074/jbc.M112.425066)
Supplement: Supplemental Data [file supp_M112.425066_jbc.M112.425066-3.doc]

**Table S1.** Identification of differentially expressed proteins by MALDI-TOF mass spectrometry in primary astrocytes derived from G93A and WT SOD1 mice and their secreted proteins.

| Spot  n. | Protein name | Acc.^a^ | Mw (kDa) calc.^b^ | pI calc.^c^ | Mw (kDa) obs.^d^ | pI obs.^e^ | Cov.^f^ | Match/ unmatch.^g^ | Score^h^ |
| --- | --- | --- | --- | --- | --- | --- | --- | --- | --- |
| **Proteins from primary astrocytes** | |  |  |  |  |  |  |  |  |
| 1 | HSP90B1 | P08113 | 92 | 4.7 | 90 | 4.5 | 17 | 12/39 | 71 |
| 2 | Filamin-A | Q8BTM8 | 281 | 5.7 | 150 | 6.2 | 19 | 31/87 | 84 |
| 3 | GRP78 | P20029 | 72 | 5.1 | 70 | 5.0 | 48 | 27/54 | 246 |
| 4 | Transketolase | P40142 | 68 | 7.2 | 70 | 7.5 | 24 | 14/45 | 101 |
| 5 | Vimentin | P20152 | 54 | 5.1 | 65 | 4.9 | 34 | 13/63 | 78 |
| 6 | PDI | P09103 | 57 | 4.8 | 60 | 4.5 | 21 | 11/40 | 79 |
| 7 | DRP-2 | O08553 | 62 | 6.0 | 60 | 5.9 | 46 | 20/52 | 153 |
| 8 | Pyruvate kinase isozyme M2 | P52480 | 58 | 7.2 | 60 | 7.5 | 52 | 24/76 | 169 |
| 9 | Mixture 1 |  |  |  |  |  |  | 49/78 | 270 |
|  | Glial fibrillary acidic protein | P03995 | 50 | 5.4 | 50 | 5.3 | 51 | 26/78 | 188 |
|  | Vimentin | P20152 | 54 | 5.1 | 50 | 5.3 | 54 | 23/78 | 164 |
| 10 | ATP synthase subunit alpha | [Q03265](http://www.matrixscience.com/cgi/protein_view.pl?file=../data/20070226/FogTSzam.dat&hit=1) | 60 | 9.2 | 57 | 8.5 | 30 | 13/58 | 82 |
| 11 | Vimentin | P20152 | 54 | 5.1 | 45 | 4.5 | 60 | 29/63 | 247 |
| 12 | Ubiquinol-cytochrome-c reductase complex core protein 1 | Q9CZ13 | 53 | 5.8 | 45 | 5.4 | 36 | 13/71 | 88 |
| 13 | RabGDI | Q61598 | 51 | 5.9 | 45 | 6.0 | 42 | 20/45 | 148 |
| 14 | Alpha-enolase | P17182 | 47 | 6.4 | 45 | 6.5 | 44 | 17/75 | 113 |
| 15 | Actin | P60710 | 42 | 5.3 | 40 | 5.5 | 31 | 10/59 | 65 |
| 16 | Erk2 | P63085 | 41 | 6.5 | 42 | 6.4 | 25 | 9/47 | 64 |
| 17 | Fructose-bisphosphate aldolase C | P05063 | 39 | 6.7 | 42 | 6.8 | 46 | 17/58 | 154 |
| 18 | Fructose-bisphosphate aldolase A | P05063 | 39 | 8.4 | 42 | 9.0 | 54 | 21/98 | 145 |
| 19 | Tropomyosin-1 alpha chain | P58771 | 33 | 4.7 | 35 | 4.5 | 44 | 14/84 | 88 |
| 20 | Tubulin beta-2C chain | P68372 | 50 | 4.8 | 28 | 5.0 | 25 | 15/62 | 76 |
| 21 | Voltage-dependent anion-selective channel protein 1 | Q60932 | 32 | 8.6 | 30 | 9.0 | 48 | 11/66 | 85 |
| 22 | 14-3-3 protein epsilon | P62259 | 29 | 4.6 | 28 | 4.4 | 52 | 15/91 | 66 |
| 23 | Actin | P60710 | 42 | 5.3 | 25 | 5.4 | 32 | 12/83 | 70 |
| 24 | Prohibitin | P67778 | 30 | 5.6 | 26 | 5.4 | 35 | 8/52 | 59 |
| 25 | Enoyl-CoA hydratase | Q8BH95 | 31 | 8.8 | 25 | 7.8 | 41 | 10/42 | 87 |
| 26 | RhoGDI | Q99PT1 | 23 | 5.2 | 23 | 5.0 | 57 | 13/66 | 103 |
| 27 | Cryab | P23927 | 20 | 6.8 | 20 | 7.0 | 45 | 9/59 | 67 |
| 28 | CypA | P17742 | 18 | 7.7 | 18 | 8.5 | 51 | 9/72 | 61 |
| **Secreted proteins from primary astrocytes** | |  |  |  |  |  |  |  |  |
| 1 | VCP/p97 | Q01853 | 89 | 5.1 | 90 | 5.0 | 17 | 14/46 | 68 |
| 2 | DRP-2 | O08553 | 62 | 5.9 | 55 | 6 | 45 | 22/58 | 173 |
| 3 | ERp57 | P27773 | 57 | 5.9 | 60 | 6.0 | 54 | 27/73 | 200 |
| 4 | SPARC | P07214 | 34 | 4.8 | 42 | 4.5 | 28 | 11/50 | 62 |
| 5 | Ubiquitin carboxyl-terminal hydrolase isozyme L1 | Q9R0P9 | 25 | 5.1 | 24 | 5.0 | 39 | 8/44 | 71 |
| 6 | h_SOD1 | P00441 | 16 | 5.7 | 17 | 6.0 | 44 | 8/20 | 114 |

^a^ Accession number from UniProtKB database; ^b^Calculated molecular weight; ^c^Calculated pI; ^d^Observed molecular weight; ^e^Observed pI; ^f^Percentage of sequence coverage; ^g^Peptide matched/peptide unmatched; ^h^Mascot score.
